# Supplementary material for: Perceptions, awareness on snakebite envenoming among the tribal community and health care providers of Dahanu block, Palghar District in Maharashtra, India
Source: PLoS One. 2021 Aug 5;16(8):e0255657. doi: 10.1371/journal.pone.0255657 (PMC8341635; doi:10.1371/journal.pone.0255657)
Supplement: S1 Table — (DOCX) [file pone.0255657.s002.docx]

**S1 Table:** Snakebite experience of peripheral health care providers in Dahanu block

| **Questions asked** | **Nurse^$^**  **N=38 (%)** | **ASHA***  **N=35 (%)** | **ANM ^#^ N=11(%)** | **MPW ^&^ N=12 (%)** |
| --- | --- | --- | --- | --- |
| How long have you been in this job?  <10 years  >10 years | 19 (50) | 11 (31.4) | 8 (72.7) | 3 (25) |
|  | 19 (50) | 24 (68.5) | 3 (27.2) | 9 (75) |
| How long have you been working here at this health facility? 0-10 Years  >10 years |  |  |  |  |
|  | 23(60.5) | 11(31.4) | 4(36.3) | 12(100) |
|  | 15 (39.4) | 24 (68.5) | 7 (63.6) | 0 (0) |
|  |  |  |  |  |
| How many snakebite cases have you seen in and around your area in the last 6 months? >10 cases  10 to 25 Cases  25 to 50 Cases  Not seen |  |  |  |  |
|  | 10 (26.3) | 20 (57.1) | 7 (63.6) | 9 (75) |
|  | 10 (26.3) | 2 (5.7) | 1 (9) | 1 (8.3) |
|  | 15 (39.4) | 0 (0) | 0 (0) | 0 (0) |
|  | 3 (7.8) | 13 (37.1) | 3 (27.2) | 2 (16.6) |

^$^ Nursing staff workers under Rural Hospital (RH) and Sub District Hospital (SDH).

^*^ Accredited Social Health Activist (ASHA); works within village and report to sub-centers

^#^ Auxiliary Nurse Midwifery (ANM); Works under sub-centers and Primary Health Centers.

^&^Multi-Purpose Worker (MPW); Works under sub-centers.
