# Supplementary material for: Perceptions, awareness on snakebite envenoming among the tribal community and health care providers of Dahanu block, Palghar District in Maharashtra, India
Source: PLoS One. 2021 Aug 5;16(8):e0255657. doi: 10.1371/journal.pone.0255657 (PMC8341635; doi:10.1371/journal.pone.0255657)
Supplement: S2 Table — (DOCX) [file pone.0255657.s003.docx]

**S2 Table:**  Demographic details and awareness of snakebite amongst traditional faith healers, and snake rescuers

| **Questions asked** | **Traditional faith healers**  **N= 9 (%)** | **Snake rescuer**  **N=12 (%)** |
| --- | --- | --- |
| Education Below 5^th^ class  Below 10^th^ class  Above 10^th^  Uneducated | 0 (0) | 3 (25) |
|  | 6 (66.6) | 1 (8.3) |
|  | 3 (33.3) | 2 (16.6) |
|  | 0 (0) | 6 (50) |
| Basic Occupation Farming  Local business  Private  Sole traditional faith healer  No specific | 2 (22.2) | 1 (8.3) |
|  | 0 (0) | 5 (41.6) |
|  | 1(11.1) | 3 (25) |
|  | 6 (66.6) | 0 |
|  | 0 (0) | 3 (25) |
| How long have you been in this service (Treating of Snakebite cases/rescue of snakes)? <10 Years  >10 years |  |  |
|  | 6 (66.6) | 4 (33.3) |
|  | 3 (33.3) | 8 (66.6) |
|  |  |  |
| How long have you been working here in this area?  <10 Years  >10 years |  |  |
|  | 3 (33.3) | 7(58.3) |
|  | 6 (66.3) | 5 (41.6) |
| How many snakebite cases you have seen in and around your area in the last 6 months?  <10  10 to 25  25-50  >50 |  |  |
|  |  |  |
|  | 3 (33.3) | 0 |
|  | 1 (11.1) | 0 |
|  | 4 (44.4) | 0 |
|  | 1 (11.1) | 12 (100) |
|  | 9 (100) | 7 (58.3) |
| Which season you have observed more no. of snakebite cases at your locality? June to September  October to December  January to May |  |  |
|  | 2 (22.2) | 0 |
|  | 7 (77.7) | 12 (100) |
|  | 0 | 0 |
