# Supplementary material for: Perceptions, awareness on snakebite envenoming among the tribal community and health care providers of Dahanu block, Palghar District in Maharashtra, India
Source: PLoS One. 2021 Aug 5;16(8):e0255657. doi: 10.1371/journal.pone.0255657 (PMC8341635; doi:10.1371/journal.pone.0255657)
Supplement: S3 Table — (DOCX) [file pone.0255657.s004.docx]

**S3 Table:** Pre and Post Training evaluation of Medical Officers

| **Knowledge areas** | **Pre-test**  **(n= 40) (%)** | **Post-test**  **(n=39) (%)** | ***p* value** |
| --- | --- | --- | --- |
| **Knowledge about snakes and symptoms** |  |  |  |
| Most of the snakes in India are non-venomous (True) | 30(75) | 30 (76.9) | 0.84 |
| The venomous snake’s head is usually oval-shaped, with regular teeth marks (False) | 22 (55) | 22 (56.4) | 0.9 |
| Which of the following are high-incidence periods of snakebite in your locality (Night time, after rain, & Summer) | 20 (50) | 24 (61.5) | 0.31 |
| What are the symptoms of snakebite (Local Bleeding, swelling, nausea, vomiting dizziness) | 34 (85) | 34 (87.1) | 0.78 |
| Local Pain/ Tissue Damage is not observed in which snakebite (Kraits) | 22 (55) | 24 (61.5) | 0.56 |
| Renal Complications is usually observed in which snakebite envenoming (Russells Viper) | 18 (45) | 30 (76.9) | 0.004 |
| **Anti-snake venom and its usage** |  |  |  |
| Anti-snake venom in India (Polyvalent) | 22 (55) | 22 (56.4) | 0.91 |
| Mechanism/functions of action of ASV (Neutralizing venom) | 19 (47.5) | 20 (53.8) | 0.58 |
| Ideal route of administration of ASV in patients with snakebite (IV with NS) | 34 (85) | 35 (89.7) | 0.53 |
| Amount of ASV dosage in pregnancy (Same as other victims) | 28 (70) | 30 (76.9) | 0.49 |
| Pediatric ASV Dosage (Same as adults) | 24 (60) | 30 (76.9) | 0.11 |
| **Laboratory investigations and first-aid practices** |  |  |  |
| How much time do you keep a victim under observation before discharge in case of non-venomous bite (24 hrs.) | 21 (52.5) | 22 (56.4) | 0.73 |
| Snakebite treatment mainly depends on (A type of snake, amount of venom, clinical picture, age of the victim) | 16 (40) | 17 (43.5) | 0.75 |
| What are the essential tests to be done for confirmed snakebite patients? (BT/CT) | 16 (40) | 18 (46.2) | 0.58 |
| First aid for snakebite should include which of the following (Immobilize the bitten part, remain calm, call emergency services) | 28 (70) | 30 (76.9) | 0.49 |
| Snake venom or components or ASV can cross the placenta (unclear) | 11 (27.5) | 12 (30.7) | 0.76 |
