# Supplementary material for: Perceptions, awareness on snakebite envenoming among the tribal community and health care providers of Dahanu block, Palghar District in Maharashtra, India
Source: PLoS One. 2021 Aug 5;16(8):e0255657. doi: 10.1371/journal.pone.0255657 (PMC8341635; doi:10.1371/journal.pone.0255657)
Supplement: S1 Text — (DOCX) [file pone.0255657.s005.docx]

**S1 Text:** Consolidated criteria for reporting qualitative studies (COREQ): 32-item checklist

Developed from: Tong A, Sainsbury P, Craig J. Consolidated criteria for reporting qualitative research (COREQ): a 32-item checklist for interviews and focus groups. International Journal for Quality in Health Care. 2007. Volume 19, Number 6: pp. 349 – 357

| **No** | **Item** | **Guide questions/description** | **Reported on Page #** | |
| --- | --- | --- | --- | --- |
| **Domain 1: Research team and reflexivity** | | |  | |
| Personal Characteristics | | |  | |
| 1. | Interviewer/facilitator | Which author/s conducted the interview or focus group? | Dr Dipak Abnave  Mr Ujwal Pachalkar  Mr Sandip Tarukar | |
| 2. | Credentials | What were the researcher's credentials? *E.g. PhD, MD* | Dr Dipak Abnave, MSW, M.Phil, Ph.D. (Sociology)  Mr Ujwal Pachalkar, Masters in Social Work (MSW)  Mr Sandip Tarukar, Masters in Social Work (MSW) | |
| 3. | Occupation | What was their occupation at the time of the study? | Dr Dipak Abnave, Scientist B (Social Scientist)  Mr Ujwal Pachalkar, Medical Social Worker  Mr Sandip Tarukar, Medical Social Worker | |
| 4. | Gender | Was the researcher male or female? | Male | |
| 5. | Experience and training | What experience or training did the researcher have? | Methods  The researcher (Social Scientist) had prior experience of qualitative research during M.Phill and PhD course at Jawaharlal Nehru University, New Delhi, India.  Training was provided to the Medical Social Workers prior to data collection. | |
| Relationship with participants | | |  | |
| 6. | Relationship established | Was a relationship established prior to study commencement? | No | |
| 7. | Participant knowledge of the interviewer | What did the participants know about the researcher? e*.g. personal goals, reasons for doing the research* | Participant Information Sheet and Informed Consent Form | |
| 8. | Interviewer characteristics | What characteristics were reported about the interviewer/facilitator? e.g. *Bias, assumptions, reasons and interests in the research topic* | Under the methods section,  Manuscript page no. 08, Line number: 160-161 | |
| **Domain 2: Study design** | | |  | |
| Theoretical framework | | |  | |
| 9. | Methodological orientation and Theory | What methodological orientation was stated to underpin the study? *e.g. grounded theory, discourse analysis, ethnography, phenomenology, content analysis* | Under the methods section,  Manuscript page no. 07, Line number: 149 | |
| Participant selection | |  |  | |
| 10. | Sampling | How were participants selected? *e.g. purposive, convenience, consecutive, snowball* | Under the methods section,  Manuscript page no.09 and 10, Line numbers:184,226 | |
| 11. | Method of approach | How were participants approached? e*.g. face-to-face, telephone, mail, email* | Under the methods section,  Manuscript page no. 09, Line numbers: 191-194 | |
| 12. | Sample size | How many participants were in the study? | Under recruitment, sampling & data collection section,  For FGD and Interviews (Manuscript page no.10 & 11, Line numbers: 211, 227), For Pre-Post testing MOs (Page no. 11, Line 243). | |
| 13. | Non-participation | How many people refused to participate or dropped out? Reasons? | Under the methods section,  Manuscript page no.10, Line number: 214-215. | |
| Setting |  |  |  | |
| 14. | Setting of data collection | Where was the data collected? e*.g. home, clinic, workplace* | Under recruitment, sampling & data collection section, Manuscript page no. 9, Line number: 189-191.  Page no. 10, Line no. 224-225 | |
| 15. | Presence of non-participants | Was anyone else present besides the participants and researchers? | No | |
| 16. | Description of sample | What are the important characteristics of the sample? *e.g. demographic data, date* | Under the methods section,  Manuscript page no. 10, Line number: 215-220, Table S1 Table, S2 Table | |
| Data collection | |  |  | |
| 17. | Interview guide | Were questions, prompts, guides provided by the authors? Was it pilot tested? | Under recruitment, sampling & data collection section Manuscript page no.10, Line number: 217-218. | |
| 18. | Repeat interviews | Were repeat interviews carried out? If yes, how many? | No | |
| 19. | Audio/visual recording | Did the research use audio or visual recording to collect the data? | Under recruitment, sampling & data collection section, Manuscript page no. 9, Line number: 199-201 | |
| 20. | Field notes | Were field notes made during and/or after the interview or focus group? | Under recruitment, sampling & data collection section,  Manuscript page no.09, Line number:201-204 | |
| 21. | Duration | What was the duration of the interviews or focus group? | Under recruitment, sampling & data collection section,  Manuscript page no.09, Line number: 185-186 | |
| 22. | Data saturation | Was data saturation discussed? | No | |
| 23. | Transcripts returned | Were transcripts returned to participants for comment and/or correction? | No | |
| **Domain 3: analysis and findings** | | | | |
| Data analysis | |  | |  |
| 24. | Number of data coders | How many data coders coded the data? | | Under data analysis.  Number of data coders - Two,  Page no. 12, Line no. 264. |
| 25. | Description of the coding tree | Did authors provide a description of the coding tree? | | Under Methods,  Manuscript page no.08, Line number: 179-181 |
| 26. | Derivation of themes | Were themes identified in advance or derived from the data? | | Under Methods,  Manuscript page no.08, Line number: 179-181 |
| 27. | Software | What software, if applicable, was used to manage the data? | | Manual coding was done. No software was used for analysis. |
| 28. | Participant checking | Did participants provide feedback on the findings? | | No |
| Reporting |  |  | |  |
| 29. | Quotations presented | Were participant quotations presented to illustrate the themes / findings? Was each quotation identified? e*.g. participant number* | | Under the Results section,  Manuscript page no.13-19, Line number: 278-402. |
| 30. | Data and findings consistent | Was there consistency between the data presented and the findings? | | Yes, Described in the discussion section  Manuscript page no.21-25 |
| 31. | Clarity of major themes | Were major themes clearly presented in the findings? | | Yes, Under Results section,  Manuscript page no.13-19 |
| 32. | Clarity of minor themes | Is there a description of diverse cases or discussion of minor themes? | | Yes, under Results section  Manuscript page no.13-19 |
