# Supplementary material for: Perceptions, awareness on snakebite envenoming among the tribal community and health care providers of Dahanu block, Palghar District in Maharashtra, India
Source: PLoS One. 2021 Aug 5;16(8):e0255657. doi: 10.1371/journal.pone.0255657 (PMC8341635; doi:10.1371/journal.pone.0255657)
Supplement: S2 Text — (DOCX) [file pone.0255657.s006.docx]

**Focus Group Discussion (FGD) Guideline**

- Total 18 FGDs: one each for male and female covering 9 PHCs in Dahanu block
- Number of participants in each group will be about 8 to 16
- Plan the suitable date and time for FGDs
- Contact the peripheral health workers and community leaders in 9 PHCs for inviting the individuals to participate in Male and Female FGD Groups
- Wellcome the participants, introduction of the Supervisor, Medical Social Workers and participants
- Provide information on purpose of study and the FGD, provide copy of Participant information sheet and informed consent form
- Supervisor to obtain written informed consent and thank participants for agreeing to participate in discussion
- MSWs to write notes and turn on the audio recorder, monitor the time (session should not be more than 60 minutes)

**Theme 1:- Awareness and knowledge about snakes and snakebite**

*Sample questions*

- What are the common snakes found in your area and their living and hiding places?
  - Are you aware of poisonous and non-poisonous snakes? Are you able to identify the poisonous and non-poisonous snake by seeing the snake?
  - What time of year and what time of day or night are these snakes likely to be out and active?

**Theme 2:- Perception and health seeking behaviour about snakebites**

*Sample questions*

- - What are the beliefs related to snakebites?
  - Are you aware of precautions to be taken to avoid a snakebite? If yes, what are they and do you follow the precautions?
  - Are you aware why snakes bite human?
  - What do you do after knowing that was snake bite? Where will you take the patient for treatment?
  - Do you visit the tantric/ temples when there is a case of snakebite?

**Theme 3:- Awareness about first aid procedures for snakebite**

*Sample questions*

- What do you do when you come across a case of snakebite?
- Are you aware of the first aid measures for a snakebite patient?
- Have you ever given first aid treatment to any patient with snakebite in your area? If so, how do you manage a patient?
- Are you aware of local ambulance services at your area? If ambulance is not available how do you transport the patient to the nearest health care facility?
