# Supplementary material for: Perceptions, awareness on snakebite envenoming among the tribal community and health care providers of Dahanu block, Palghar District in Maharashtra, India
Source: PLoS One. 2021 Aug 5;16(8):e0255657. doi: 10.1371/journal.pone.0255657 (PMC8341635; doi:10.1371/journal.pone.0255657)
Supplement: Attachment — Submitted filename: Rebuttal letter.docx [file pone.0255657.s007.docx]

Prof. Joerg Heber

Editor-in-Chief, PLOS ONE

1160 Battery Street, Koshland Building East, Suite 225

San Francisco, CA 94111

United States.

**Subject**: Point by point response to reviewers and editors comments for the manuscript “Perceptions, awareness on snakebite envenomation among the tribal community and health care providers of Dahanu block, Palghar district in Maharashtra, India”

Dear Sir,

We thank the reviewers and editors for their valuable time, critical inputs and useful suggestions for our manuscript. We appreciate their inputs to improve our manuscript. Following are our point by point response to reviewers and editors comments.

**RESPONSE TO REVIEWER’S COMMENTS PLOS NTD**

**Reviewer # 1**

**General Comments**

**Reviewer #1:** The objectives of the study are clear, and the population is clearly stated.

-Non statistical analysis was done.

-The population is adequate.

No new analysis is required.

-Some details have to be addressed (see comments).

**Reviewer #1:** -The analysis matches the plan.

-The result are completely presented; However the results can be explained better (optional) by using charts, for instance.

Authors Must Improve quality of figures and tables.

Reviewer #1: The conclusion are supported by the data.

-Authors show limitations; however, they have to discuss more.

-Authors discuss the benefit of their work.

-Yes.

**Reviewer #1:** Please review use of commas, subject-object agreement, punctuation marks, etc.

**Response:** We wish to sincerely thank reviewer for the critical review of our manuscript and providing valuable suggestions for improving our manuscript. The section on limitations is discussed in more details as follows in the revised manuscript, page nos. 26-27, line nos. 582-602.

*Limitations:*

To the best of our knowledge, this is the first study that contributed to evidence on preventive measures and awareness of snakebite envenomation among the tribal community and health care providers in Maharashtra India. However, the study has limitations. Firstly, the study was carried out in Dahanu block of Palghar District which may not be representative of the total tribal population in Maharashtra state. Thus, perceptions and awareness about snakebite could be region specific and may not be representative of the entire population in the State. Secondly, all FGD and in depth interviews were conducted in the vernacular language (Marathi) and later translated into English. Local snake rescuers were involved in translations and interpretations of some of the words used by tribal communities during FGDs. Hence, some interpretations may have been lost in translation. We did not record the age of the FGD participants missing out age wise data analysis. However, we tried to include participants reflecting a wide range of ages, community leaders and members, older and younger to minimize the recruitment bias. The information on how the traditional healers treat snakebites could not be obtained which is one of the limitations of the study. The pre and post-test to assess the knowledge and management practices were carried out just before commencement of training and then repeated immediately after the training of Medical officers. Therefore, results of pre and post training survey might reflect what was taught/discussed during the training. However, these Medical Officers are working at different levels of public health care facilities (PHCs, RH and SDHs) in the Dahanu block and most of them are actively involved in the management of snakebite cases at their respective public health hospitals. Repeated training programs are conducted and Standard Treatment Guidelines (STD,2017) of Government of India is implemented in the study area.

In the revised manuscript, we tried our best to take care of the use of commas, subject-object agreement, punctuation marks mistakes and other editorial mistakes. As per the suggestions, we have presented results using charts ( Figure 3, Figure 4).

**Specific Comments:**

Reviewer #1: Some comments have to be addressed.

**Line 53.- Please review and add information: Vulnerability to snakebite envenoming: a global mapping of hotspots. Long bottom, Joshua et al. The Lancet, Volume 392, Issue 10148, 673 – 684.**

**Response:** We thank the reviewer for suggesting this important article. As suggested we have added the information in the discussion and revised the following sentence in the abstract :

A multi-sectoral approach which includes community awareness, mapping of vulnerable populations to the most severe outcomes of snakebite envenoming, capacity building of local health care facility, empowerment of Medical Officers and other healthcare providers on management of snakebite could be useful for reducing the mortality and morbidity due to snakebite envenomation in India as well as other tropical countries.

The changes are incorporated in the revised manuscript, page no. 3 line nos. 53-57.

Following para is revised in the discussion section, Page no. 28, line nos. 617-626.

A global mapping of hotspots identified the most vulnerable populations to 278 medically important snake species for the most severe outcomes of snakebite envenoming [61]. Bases on these observations and our experience from the present study, we propose a multi-sectoral model which includes community education, mapping of vulnerable populations to the most severe outcomes of snakebite envenoming, capacity building of local health care facility, empowerment of Medical Officers and healthcare providers on management of snakebite as national snakebite protocol (STG,2017) transdisciplinary expertise including animal health, herpetology, forestry, anthropology, and education (Figure 5).

**Line 53.- How education could reduce morbidity? Indicate otherwise edit.**

**Response:** We agree with the reviewer and the word ‘education’ is replaced by ‘awareness’. In the revised manuscript Page no. 3, line 53.

**Line 103.- could you please mention what snake antivenoms are available in India?**

**Response:** At present, polyvalent ASV raised in horses using venoms of four important venomous snakes; Indian cobra (*Naja naja*), Indian Krait (*Bangarus Caeruleus*), Russell’s viper (*Daboia russelii*) and saw-scaled viper (*Echi carinatus*) is the only venom-specific life-saving effective treatment available In India [7]. This information is included in the revised manuscript, page no. 4, line no. 91-94

**Line 103.- could you please mention why intradermal ASV test is irrational? What do you mean with Intradermal ASV test? Please clarify.**

**Response:** By intradermal ASV test, we mean the skin hypersensitivity testing of ASV, which is carried out by intradermal injection of 0.1ml of ASV. Skin hypersensitivity testing of ASV is carried out by intradermal injection of 0.1ml of ASV. However, this intradermal ASV testing is non-predictive as ASV reactions are mediated by direct activation of complement system and not mediated by IgE [2,3]. Intradermal skin testing may delay treatment when patient needs ASV, and it may also pre-sensitize the patient leading to more severe reaction in case a large amount of ASV is to be administered [4]. Therefore, National and International Snakebite Management Protocols[4–6] strictly recommended that ASV test dose should not be administered. In spite of these National and International guidelines, there is irrational usage of ASV intradermal skin testing in Maharashtra [7,8].

We included information in revised manuscript, page no. 5, line nos. 94-102

**Line 107.- in your phrase: “Recently, Indian cobra (Naja naja) genome has been sequenced and 19 key toxin genes have been identified in venom gland. This will help in targeting the proteins responsible for toxic effect and antidotes to neutralize their activity could be developed which will act as an anti-venom. could you explain how or in which way? A possibility is by targeting only key toxins. see: de la Rosa, G., Olvera, F., Archundia, I.G. et al. Horse immunization with short-chain consensus α-neurotoxin generates antibodies against broad spectrum of elapid venomous species. Nat Commun 10, 3642 (2019).** [**https://doi.org/10.1038/s41467-019-11639-2**](https://doi.org/10.1038/s41467-019-11639-2)

**Response:** We have reframed the sentence as per the comment, page no. 5, Line nos. 103-105

Recently, Indian cobra (Naja naja) genome has been sequenced and 19 key toxin genes have been identified in venom gland. Neutralization of these key toxins using antibodies could be useful for developing an effective therapeutic strategy [15].

**Line 108.- if they are not treated in public health facilities, where are they treated? Please indicate.**

**Response:** The snakebite victims especially in rural areas visit the traditional faith healers and hence not all snakebite victims are treated in public health facilities. The sentence is reframed as follows, page no. 5, line nos. 105-108.

The morbidity and mortality of snakebites are usually underreported especially in rural areas as majority of the victims first report to traditional healers [16] and hence not all snakebite victims are treated in public health facilities[17][18].

**Line 110. 1.3 M? Please cite the corresponding source, edit or rephrase.**

**Response:** As suggested by the reviewer, we have edited the sentence as follows :

The mortality due to snakebite envenoming varies from 81,410 to 137,880 per year worldwide [19,20]. Page no. 5, line nos. 109-110.

**Line 122. please clarify….24.9/100,000 person-years. What does it mean?**

**Response:** It means in the community if we follow 100,000 persons for 1 year or 50,000 persons for 2 years or 200,000 persons for 6 months, 24.9 will have envenomation.

**Line 124. 7.23% of deaths by snake where reported? what happened to the remaining ~92.77%?**

**…22.19 % where referred to public hospitals. what happened to the rest? are these estimates or real numbers? Please cite your source and clarify.**

**Response:**

A community based epidemiological survey was conducted in eight blocks of the South 24-Paraganas district of West Bengal, India from January 2009 to October 2010. The data of this direct survey was then compared with the official report of the same area to find out the under reporting of snakebite deaths in health facilities in that area during the study period. The authors reported that there was a huge gap between their direct survey data and official data of the same area. From the survey, the authors found that the total number of snakebite cases were 4871 and snakebite deaths were 184 in 2 years. Only 7.23% of snakebite deaths were officially reported and the rest of the deaths were not reported in the government official records as revealed from the findings of the study.

In this study, the authors reported that only 22.19% of snakebite patients attended local government hospitals. Out of the rest 77.8%, a small percentage tried to go to the sub-divisional hospitals, but majority went to the local traditional faith healers for snakebite treatment. These are the real numbers obtained from the door-to door survey. The reference has been cited in the revised manuscript, Page no. 6, line nos.118-122.

**Line 126-128.- Please rephrase. It is understood by the phrase that 80 % and 40 % = 120 %. Please make this clear.**

**Response :** We thank the reviewer for bringing out our mistake. We have rephrased the sentence as follows (page no. 6, line nos. 122-123) :

In a neighboring country Nepal, 80% of snakebite deaths occurred either in villages or during transport to the health care facility and only 20% of deaths occurred in a hospital [25].

**Line 162.- Please indicate the approval number or approval code.**

**Response :** Institutional Ethics Committee approval number (D/ICEC/Sci-108/145/2016) has been added in the revised manuscript, page no. 7, line no. 157.

**Line 254 Please indicate why the age of the participant was not taken and what was the range of ages among participants regardless gender. Common sense would indicate that older people would have a higher degree of awareness compare with younger people. Is this a bias in your work?**

**Response:** We understand the importance of age of the participant for FGD. However, in the trial focus group discussions especially in women, it was observed that the tribal community is reluctant to share the age in front of other community members from their village. Therefore, we did not record age of the participants. The focus group participants were mobilized with the help of peripheral health care workers of the village. The range of ages among participants regardless of gender were approximately from 18 to 60 years. A total of 202 participants [ Males 90 (44.5%) and Females 112 (55.4%)] participated in a total of 18 FGDs in 9 PHCs in the study block. We tried to include participants reflecting a wide range of ages, community leaders and members, older and younger to avoid any bias.

**Line 264.- When you state that people identify venomous snake by color…. bite marks and color bands… does it mean that they can identify even the species, or just to separate venomous from non-venomous?**

**Response:** Community members during FGD mentioned that they can identify some of the species of venomous snakes like Naja Naja and Viper based on color, size, shape, tail, bite marks.

**Table 1.- S. No 5. What do you mean by semi-venomous? To the best of my knowledge they are venomous and non-venomous snakes.**

**Response :**	In India, semi-venomous snakes are also observed. These semi-venomous snakes are classified as “mildly venomous”. We have edited the term from semi-venomous to mildly venomous. Mildly venomous snakebites are not fatal for humans. These snakes usually induce a slight numbness or tingling sensation, and it may occasionally trigger a severe allergic reaction.

List of mildly venomous snakes in India is available in the following link; <http://indiansnakes.org/>

**Line 344. A great piece of information would be how the healers treat snakebites. What traditional beverages or remedies they use? I think this very informative. e.g see: Jean-Philippe Chippaux, Ismaila Diédhiou, Roberto Stock . Étude de l’action de la pierre noire sur l’envenimation expérimentale. Cahiers d'études et de recherches francophones / Santé. 2007;17(3):127-131. doi:10.1684/san.**2007**.0076.**

**Response :** We agree with the reviewer that information on how the traditional healers treat snakebites is of great importance. During the face to face interview, we tried to get the information on what traditional methods/medicines they use for treating snakebite patients. In the present study, we conducted Face to face in-depth key informant interviews with 09 traditional faith healers. None of the traditional healers were ready to share the formulation with anyone. They believed that, if they share the information on what traditional methods/medicines they use for treating snakebite patients, their method won't work in future. This information has been added to the revised manuscript page no. 16-17, line nos. 363-366.

As suggested by the reviewer, we have quoted the suggested article in the discussion part in the revised manuscript page no.21, line no. 477-483.

Although there are reports on use of snakestone (black stone) to treat snakebites since ancient times, there is negligible scientific literature on its therapeutic efficacy. Chippaux *et al.* conducted a study to determine therapeutic efficacy of black stones using animal model but failed to show any therapeutic efficacy [35]. Since traditional remedies do not have any proven benefit in treating snakebite, it is recommended to avoid traditional first aid methods including black stones and alternative medical/herbal therapy [10].

**Line 361.- Table 3.- 9 healers and 12 Sarpamitras. Why in some sections the total of either healers or sarpamitras is not equal to the total N? for instance, in education section only 9 Sarpamitras . please clarify.**

**Response:** During the study period, we could enroll only 9 traditional faith healers and 12 snake rescuers as per their availability and willingness to participate in the study. A typographical error occurred in the educational section mentioned in Table 3. We have corrected this error in revised manuscript, Table S2.

**Why you mentioned Dopamine, frusemide, neostigmine and Epinephrine? Please indicate, the therapeutic use of these drugs.**

**Response:** Dopamine is a vasopressor drug required for treating hypotension and shock that persist after antivenom treatment. If the patient doesn’t respond to fluid resuscitation, inotropic support with dopamine is recommended. In case of vasculotoxic snakebite, for forced alkaline diuresis (FAD) Inj. Frusemide is recommended along with other drugs. Neuroparalytic Snakebite cases require intensive monitoring and may requires assisted life support. Primary and secondary health care centers without ICU support are recommended to refer the patient in an advance life support ambulance to the higher center immediately after giving Atropine – Neostigmine injection. Atropine-Neostigmine (an acetylcholinesterase inhibitor) are indicated for improvement of neuromuscular transmission temporarily. Epinephrine (Adrenaline) is a lifesaving drug required for management of anaphylactic reaction due to ASV. Availability of these drugs at public health care facilities is strongly recommended by National Snakebite Management protocol (2017) of Government of India (file:///D:/Snakebite%20Project%20National%20_ICMR%202019/Management%20of%20Snake%20Bite,%20GOI%202017.pdf) .

**Line 467. Develop more the importance of awareness and prevention. You already cite “Snakebite envenoming - A strategy for prevention and control” published by WHO. Please discuss more. The same for line 498-500**

**https://www.who.int/snakebites/resources/9789241515641/en/**

**Response:** As suggested, we have included the information on importance of awareness and prevention in light of the recent WHO strategy document. Following information is added in discussion section of the revised manuscript, page no.20, line nos. 446-469.

To the best of our knowledge, this is the first study to investigate knowledge, awareness, health-seeking practices, first aid, and prevention of snakebites in one of the largest state of Maharashtra, India. Although most of the participants were aware of snakes and their bites, many of them were not able to identify snakes by local names or by watching snakes. The community members were able to recognize whether a particular snake is venomous or non-venomous after the bite based on their different perceptions about snakes and snakebite management. These observations of inability of the community to correctly identify venomous or non-venomous snakes or their claim of being able to recognize particular snake were similar to the report from USA [36]. If the community identify non-venomous snakes as venomous, this would increase the risk of and vulnerability of tribal population to snakebite envenomation. Accurate identification of venomous and non-venomous species of snakes would not only be useful for prevention of snakebite but also minimizes the chances of multiple bites to a snakebite victim or a bite to the first aid provider [37]. Therefore, knowledge and awareness of venomous and non-venomous snakes in the locality, there living and hiding places, seasons and timings when snakes are active in residential or visiting area, perceptions about snakes and snakebites of venomous snakes are of paramount importance. Preventive measures such as wearing of long shoes with enclosed toes (rather than sandals)[28], use of mosquito net on a bamboo cot or bed above the ground level could protect from krait and cobra bites [28,38–40]. Challenges in implementation of preventive measures are reported both amongst illiterate and highly educated class of the society [37] indicating need of education about snakes and snakebite prevention right from the school level to the university [41]. WHO strategy for prevention and control of snakebite envenomation focuses on prevention of snakebite, provision of safe and effective treatment, strengthening health systems, and increased partnerships, coordination, and resources [42,43].

Page no.24, for line 539-546, we have expanded the discussion as follows :

In the study area of Dahanu, domestic animals including cattle, goats, horses, sheep are an important part of the livelihoods of tribal communities. Globally, around 50 % of the poor population is completely or partially dependent on livestock for their livelihoods[61].These domestic animals are affected by venomous snakes, causing fatality rates of more than 47% in livestock [50]. Therefore, active engagement with communities to create awareness on prevention of human snakebites may also increase their awareness of snakebites among domestic animals [2]. The WHO strategy on prevention and control recommends a model of “One health” which includes collaborations between human and veterinary healthcare system.

**As a comment and questions that might be answered:**

**Why people seek local healers instead of professional assistance? What is the cost of antivenom? What is the net cost (average) of each treatment?**

**Response:** There could be several reasons for seeking treatment from traditional faith healers rather than visiting hospitals for professional assistance such as strong belief of rural and tribal communities in traditional healers and low cost for traditional healing, non-availability of doctors, shortage of ASV in public healthcare facilities, limited access of public transportation. Sometimes, medical officers are not available at public health facility especially during night hours[55] so the poor villagers are left with option of going to the locally available traditional healer. We have included this text in discussion section of revised manuscript, page no. 23, Line nos. 503-509.

In India, commercially produced ASV is relatively cheaper than ASV produced in Western countries [59]. Each vial of ASV containing 10 ml of antivenin costs about 450-550 rupees (6-8 USD). Majority of the snakebite envenomation, requires at least 10 - 20 vials of ASV and in some of the cases, higher doses up to 200 vials were reported [60]. The estimated treatment cost of snakebite treatment in private hospitals range from Rs 5,000 to 200,000 [60]. According to a study conducted in Tamilnadu, total direct costs for ASV treatment were as high as $5,700 [26]. This amounts to a huge burden to the rural and tribal poor patients working in agricultural fields who are the most common victims of snake bites. Hence, treatment of venomous snakebites at private hospitals is unaffordable to the rural and tribal populations. ASV is given free of cost in the government hospitals for snakebite treatment in India. There is a need of community awareness and sensitization to avoid delays in transferring the snakebite patients to nearest public health facility having ASV to seek medical care as life saving time would be lost. This message should be communicated correctly to the rural and tribal communities who are at maximum risk of snakebite in India.

We have included this text in discussion section of revised manuscript, page no.24, line nos. 524-538.

---------------------------------------------------------------------------------------------------------------------

**REVIEWER #2**

**Comment : The methods are not adequately defined. FGDs with the community appear to have structured questions with themes and sub-themes rather than a guide to facilitate in depth discussion about the perceptions, awareness and practices in the community at large.**

**Response**: We used FGD guide to facilitate in depth discussion about perceptions, awareness and practices in the community. As per the comemnts, we have revised the methods section as follows and included copy of FGD guide as supplementary file. We are grateful to the reviewer for critical review and valuable suggestions. The method section related to FGD is revised as follows taking into consideration reviewers comments page nos. 8- 10, line nos. 165-216.

**Study design**

This was a qualitative study conducted from June 2016 to October 2018 in Dahanu block (19.97° north and 72.73°east), of Palghar district in Maharashtra, India. Eighteen focus group discussions (FGD) were conducted from July 2017 to December 2017 to facilitate in depth discussion to understand perception, awareness and knowledge about prevention, first aid practices and healthcare seeking behavior of the community for snakebite. A FGD guide was developed and adapted by our research team (Supplementary data), all of them were proficient in vernacular Marathi language. The research team also included one MSW, native of the study area to facilitate and help the team to understand the cultural issues. As per the data of India 2011 census, total population of Dahanu Taluka is 4, 02,095, of which ~ 70 % is tribal population (Tribal 2, 77,904). There are different tribes namely; Warli, Kokana, Mahadev koli, Malhar Koli, Dhodi, Katkari in the study area with different cultural practices. Therefore, it was essential to conduct minimum 2 FGDs (one each separately for male and female) per PHC giving us a total of 18 FGDs. All 18 FGDs were carried out by the same facilitator (social scientist) with the help of two trained medical social workers to avoid any impact on the data due to the potential variation between different FGD facilitators. Themes explored in the FGD were :1) Awareness and knowledge about snakes and snakebite, 2) Perception and health seeking behavior and 3) Awareness about first aid procedures for snakebite.

**Recruitment, sampling and data collection**

FGDs were held separately for males and females for each PHC in the study area. The selection of the male and female participants for FGD were according to convenience. Each FGD had approximately 8-16 participants and participation was voluntary. Each FGD session was conducted for no longer than 60 minutes. All FGDs were facilitated by a supervisor (social scientist) who communicated with the peripheral health workers and community leaders for finalizing the date and time for FGD. The information about participation in FGD was given to the community through peripheral health workers and community leaders. Once the date was finalized, supervisor identified the venue for conducting the FGD in consultation with peripheral health workers and community leaders. He contacted the peripheral health workers and community leaders a day before the scheduled FGD for reminding them and re-confirming the participation of the invited participants from the community On the day of FGD, supervisor carried the printed consent forms in vernacular language, audio recorder, stationary and ensured the necessary logistic arrangements for conducting the FGD. Supervisor was assisted by two social workers for writing the notes and recording the discussion. After briefly introducing the participants about purpose of the focus group and a copy of participant information sheet and informed consent form in vernacular language (Marathi) was provided. After obtaining the written informed consent, facilitator assigned the responsibility to MSWs for taking notes, monitoring the audio recording and also checking the timer for keeping the track of time. With permission from the study participants, discussions of focus group were audio recorded and backed up by field notes by trained MSWs. Supervisor facilitated the discussion on perception, awareness and practices about snakebite in the community by encouraging participants to explore the discussion topics in depth and also giving the participants an opportunity to raise their own issues.

In the trial focus group discussions, it was observed that the tribal community is reluctant to share the age in front of other community members from their village. Therefore, we could not record age of the participants. However, the range of ages among participants regardless of gender was very broader from 18 to 60 years. A total of 202 participants [ Males 90 (44.5%) and Females 112 (55.4%)] participated in a total of 18 FGDs in 9 PHCs in the study block. We tried to include participants reflecting a wide range of ages, community leaders and members, older and younger to avoid any bias. Three trial focus group discussions conducted during training were excluded from the present data analysis. During the discussion, if the participant used any tribal/other than local language, these points were written and further translated with the help of local snake rescuers.

**Comment : With in-depth discussions fewer focus groups might have been sufficient; so, it is not clear what was the rationale to conduct 18 FGD. If it was to invite participants from catchment ares of each of the 9 health centres then it needs to be clearly defined with analysis and results informing about if or if not there were some differences across various areas of the study setting.**

**Response:** As per the data of India 2011 census, total population of Dahanu Taluka is 4, 02,095, of which ~ 70 % is tribal population ( Tribal 2, 77,904). There are different tribes namely Warli, Kokana, Mahadev koli, Malhar Koli, Dhodi, Katkari in the study area with different cultural practices. Therefore, we felt that it was essential to conduct minimum 2 FGDs (one each separately for male and female) per PHC giving us a total of 18 FGDs. Hence, we conducted 18 FGDs to cover the representatives of the population amongst different tribal communities residing in the study area. We did not carry out the analysis whether there were any similarities and/or differences across various areas of the study setting. The information is included in the method section of revised manuscript, page no. 8, line nos. 172-177.

**Comment: It is not clear what is meant by the term 'qualitative and cross sectional' survey'. Does it mean that the method was not a focus group discussion but a 'survey' where each participant was asked to answer a set of questionnaire which included some questions with pre-defined codes/answers and some open-ended questions?**

**Response:**

- We used qualitative study design and conducted FGDs to facilitate in depth discussion to understand awareness and knowledge about prevention, first aid practices and healthcare seeking behavior of the community for snakebite. It was a FGD and not the survey.
- Face to face in depth interviews of the healthcare providers were conducted using a pre-tested questionnaire. The different questionnaires were used for different levels of the healthcare providers. There was a separate questionnaire for traditional faith healers and snake rescuers.
- The facility check survey was conducted using separate pre-tested questionnaires for different levels of health care facilities.

We have revised the methodology section to bring out more clarity on the study design, page nos. 7-12 , line nos. 158-271.

**Comment: Method of recruitment of FGD participants needs to be explained. If the health care workers identified/encouraged the people to participate, whether some mechanism were in place to make sure that those who are in contact with the health workers on a regular basis are not over represented.**

**Response:** All FGDs were facilitated by a supervisor (social scientist) who communicated with the peripheral health workers and community leaders for finalizing the date and time for FGD. The information about participation in FGD was given to the community through peripheral health workers and community leaders. Once the date was finalized, supervisor identified the venue for conducting the FGD in consultation with peripheral health workers and community leaders. He contacted the peripheral health workers and community leaders a day before the scheduled FGD for reminding them and re-confirming the participation of the invited participants from the community. We tried to include participants reflecting a wide range of ages, community leaders and members, older and younger to avoid any bias. By contacting both the peripheral health workers and community leaders simultaneously, we could avoid overrepresentation of people who are in contact with the health workers on a regular basis. We also included participants who were not informed by healthcare worker or community leader but were eligible for participation and were available on the day of FGD.

**Comment: Recruitment for qualitative in-depth studies requires that the recruitment is strategic in order to make sure that the community members who are well aware of the perceptions, knowledge and practices of their fellow community members participate**.

**Furthermore, it is mentioned that all FGDs were conducted by medical social worker. It is not clear if one such worker conducted all FGDs or many workers were involved in conducting the interviews.**

**Similarly the role of supervising researcher needs to be described i.e. how exactly she/he supervised? Whether s/he facilitated the discussions with the medical social worker. Hence, there is need to describe the methods with clarity and detail.**

**Response:** We agree with the reviewer’s comment that recruitment for qualitative in-depth studies requires that the community members who are well aware of the perceptions, knowledge and practices of their fellow community members participate. We have included the details of the recruitment strategy in the revised manuscript. With the help of peripheral healthcare providers and community leaders in the study area, we invited and ensured participation of community members who are well aware of the perceptions, knowledge and practices of their fellow community members. All 18 FGDs were carried out by the same facilitator (social scientist) with the help of two trained medical social workers.

The recruitment strategy for FGD, exact role of supervising researcher and MSWs is included in the revised manuscript as follows. Page no. 8-10, line nos.183- 216.

**Study design**

This was a qualitative study conducted from June 2016 to October 2018 in Dahanu block (19.97° north and 72.73°east), of Palghar district in Maharashtra, India. Eighteen focus group discussions (FGD) were conducted from July 2017 to December 2017 to facilitate in depth discussion to understand perception, awareness and knowledge about prevention, first aid practices and healthcare seeking behavior of the community for snakebite. A FGD guide was developed and adapted by our research team (Supplementary data), all of them were proficient in vernacular Marathi language. The research team also included one MSW, native of the study area to facilitate and help the team to understand the cultural issues. As per the data of India 2011 census, total population of Dahanu Taluka is 4, 02,095, of which ~ 70 % is tribal population (Tribal 2, 77,904). There are different tribes namely Warli, Kokana, Mahadev koli, Malhar Koli, Dhodi, Katkari in the study area with different cultural practices. Therefore, it was essential to conduct minimum 2 FGDs (one each separately for male and female) per PHC giving us a total of 18 FGDs. All 18 FGDs were carried out by the same facilitator (social scientist) with the help of two trained medical social workers to avoid any impact on the data due to the potential variation between different FGD facilitators. Themes explored in the FGD were :1) Awareness and knowledge about snakes and snakebite, 2) Perception and health seeking behavior and 3) Awareness about first aid procedures for snakebite.

A qualitative and cross-sectional survey was carried out from June 2016to October 2018 for assessing the knowledge, awareness, prevention and first aid practices on snakebites among community. A pretested and validated questionnaire was used to assess the level of knowledge existing about venomous and nonvenomous snakes, first aid, prevention and treatment of snakebites among healthcare providers in nine Primary Health Centers (PHCs), two Sub District Hospital (SDHs) and one Rural Hospital (RH) in Dahanu block (19.97°) north and 72.73°east), of Palghar district in Maharashtra, India.

**Recruitment, sampling and data collection**

FGDs were held separately for males and females for in each PHC in the study area. . The selection of the male and female participants for FGD were according to convenience. Each FGD had approximately 8-16 participants and participation was voluntary. Each FGD session was conducted for no longer than 60 minutes. All FGDs were facilitated by a supervisor (social scientist) who communicated with the peripheral health workers and community leaders for finalizing the date and time for FGD. The information about participation in FGD was given to the community through peripheral health workers and community leaders. Once the date was finalized, supervisor identified the venue for conducting the FGD in consultation with peripheral health workers and community leaders. He contacted the peripheral health workers and community leaders a day before the scheduled FGD for reminding them and re-confirming the participation of the invited participants from the community. On the day of FGD, supervisor carried the printed consent forms in vernacular language, audio recorder, stationary and ensured the necessary logistic arrangements for conducting the FGD. Supervisor was assisted by two social workers for writing the notes and recording the discussion. After briefly introducing the participants about purpose of the focus group and a copy of participant information sheet and informed consent form in vernacular language (Marathi) was provided. After obtaining the written informed consent, facilitator assigned the responsibility to MSWs for taking notes, monitoring the audio recording and also checking the timer for keeping the track of time. With permission from the study participants, discussions of focus group were audio recorded and backed up by field notes by trained MSWs. Supervisor facilitated the discussion on perception, awareness and practices about snakebite in the community by encouraging participants to explore the discussion topics in depth and also giving the participants an opportunity to raise their own issues.

In the trial focus group discussions, it was observed that the tribal community is reluctant to share the age in front of other community members from their village. Therefore, we could not record age of the participants. However, the range of ages among participants regardless of gender was very broader from 18 to 60 years. A total of 202 participants [ Males 90 (44.5%) and Females 112 (55.4%)] participated in a total of 18 FGDs in 9 PHCs in the study block. We tried to include participants reflecting a wide range of ages, community leaders and members, older and younger to avoid any bias.

Data on age of the participants were not collected. The selection of the male and female participants for FGD were according to convenience. The focus group discussion participants were recruited with the help of peripheral health care providers of the village. Each FGD had approximately 8-16 participants. Participation was voluntary. Discussion topics included awareness, common beliefs about snake and snakebite. Three trial focus group discussions conducted during training were excluded from the present data analysis. All FGDs were carried out by a trained medical social worker (MSW) under the supervision of experienced research staff. Written informed consent was obtained from all participants before FGD. Each FGD session was conducted for no longer than 60 minutes. During each session, the initial discussion was on awareness about snakebites, beliefs, first-aid practices. Once this discussion was over, the trained MSW introduced an FGD guide developed for the study. The total FGDs were divided into 3 themes and sub/minor themes. Questions focused on the general awareness of local snakes (both venomous &nonvenomous) and the beliefs being followed. All FGDs were conducted in local language/settings (Marathi). During the discussion, if the participant used any tribal/other than local language, these points were written and further translated with the help of local snake rescuers.8

**Reviewer #2:**

The results of various sub-studies (FGDs, Interviews with health workers, with traditional health care providers, facility data, before and after training knowledge are provided in detail. However, the results lack clarity and needed detail. For example, while the main objective of the research is to gain an understanding about awareness and perceptions the section on awareness and perception is too short. Some of the data is presented as if FGD was a quantitative questionnaire The concept of FGD is to facilitate an in-depth discussion about what is the situation in the general community (as reported by the FDG participants), i.e. whether the group considers the community as being aware or not, which groups among the community are more aware etc. This is in contrast to a survey questionnaire where each respondent answer the questions about his/her own knowledge and practices.

**Response:** As per the suggestions we have revised the manuscript, page no. 12-19, line nos.272-414.

**Table 1, in that regard, does not inform if all the participants agreed about the
knowledge among the community at large.**

**Response:** We agree that Table 1 does not inform if all the participants agreed about the

knowledge among the community at large. We aim to share the information on community perception and knowledge about venomous and non-venomous snakes in the tribal block of Dahanu. Therefore, the information provided in Table 1 depicts the overall perception and knowledge about venomous and non-venomous circulating snakes in the Dahanu block and does not mean that all the participants agreed about the knowledge among the community at large. The changes are made in revised manuscript, page no. 13 , line nos. 287-289.

**Reviewer #2: The conclusion about the need for health education is based on the results. However, the limitations of analysis are not clearly described.**

**Response:** In the revised manuscript, we have described the limitations of the analysis, page nos. 26-27, line nos. 581-602.

**Specific Comments:**

**Reviewer #2: It appears that the authors conceptualized and implemented a larger, and much needed considering the lack of knowledge among communities and health care providers, project to address the snakebite knowledge, first aid and treatment in the study setting. The paper tries to sum up all the activities in one paper, and that has affected the focus of the paper and coherence of the objective-method-results. Some general concerns about the methods and results sections are mentioned above.**

**Following are some specific comments**.

**Response:** We appreciate the critical comments and suggestions of the reviewer. We are herewith clarifying the objectives of the study and method followed to achieve each objective. The overall aim of the study was to gain understanding on snakebite knowledge, first aid and treatment amongst the community and healthcare providers in tribal block of Dahanu, Maharashtra, India.

The specific objectives of the study were as follows:

1. To understand awareness and knowledge about prevention, first aid practices and healthcare seeking behavior of the community for snakebite
2. To understand the awareness, knowledge and management practices for snakebite among the traditional faith healers, snake rescuers, frontline health workers, and Medical Officers in Dahanu, Maharashtra
3. To assess ASV availability and utilization in public healthcare facilities in Dahanu block of Maharashtra
4. To provide training to the Medical Officers as per the Standard Treatment Guidelines of Government of India (STG,2017) for their capacity building on prevention, diagnosis and management of snakebite

**Objective 1 :** We used qualitative study design and conducted FGDs to facilitate in depth discussion to understand awareness and knowledge about prevention, first aid practices and healthcare seeking behavior of the community for snakebite.

**Objective 2 :** Face to face in depth interviews were conducted using a pre-tested questionnaire. The different questionnaires were used for different levels of the healthcare providers. There was a separate questionnaire for traditional faith healers and snake rescuers.

**Objective 3:** The facility check survey was conducted using separate pre-tested questionnaires for different levels of health care facilities.

**Objective 4:** Intense training was provided to the Medical Officers on prevention, diagnosis and management of snakebite as per the Standard Treatment Guidelines. Pre and post training test was conducted using a self-administered questionnaire for assessing the knowledge prior to training and improvement after training. These Medical Officers are working in the public health care facilities in the Dahanu block and were actively involved in the management of snakebite cases at their respective public health hospitals. Repeated training programs were conducted for the Medical Officers to assess knowledge retention and improvement in management practices. Since snakebite is a major burden in the study area, capacity building of healthcare providers was also part of our study.

Our aim was to give complete information related the work carried out in the tribal community resulting into a model for replicating in other areas. Therefore, we included the information of all activities in the same manuscript. In the revised manuscript we tried to reframe the text with more clarity in light of the reviewer’s comments.

**ABSTRACT: It is not clear what is meant by ‘we explored the awareness of the public health care system …” ? was it the awareness about the health services/Anti venom availability etc among the respondents?. Similarly, not well described what is meant by ‘we also focused on the capacity building of medical officer …."**

**Response:** The sentence ‘we explored the awareness of the public health care system …” is revised as “ The aim of the study was to gain understanding on snakebite knowledge, first aid and treatment amongst the community and healthcare providers in tribal block of Dahanu, Maharashtra, India”. Page no. 2, line nos. 28-31.

The sentence ‘we also focused on the capacity building of medical officer …." is modified as The effectiveness of training on snakebite was also assessed. Page no. 2, line nos. 30-31.

**In the title and at other places the word 'Perceptions' is mentioned. There is a need to describe 'perception about what'.**

**Response:** We agree with the reviewer suggestion. The perception is about snakes and snakebite management. As suggested we have revised sentence wherever the word perception is used.

**Methods are not written adequately. The terms FGD, Qualitative, Cross Sectional Survey are mentioned for probably the one method. Need to explain in detail whether it was a discussion or if the participants were asked to complete a questionnaire.**

**Response :**.

- We conducted FGDs to facilitate in depth discussion to understand awareness and knowledge about prevention, first aid practices and healthcare seeking behavior of the community for snakebite.
- Face to face in depth interviews of the traditional faith healers, snake rescuers and healthcare providers were conducted using a pre-tested questionnaire. The different questionnaires were used for different levels of the healthcare providers.
- The facility check survey was conducted using separate pre-tested questionnaires for different levels of health care facilities.

We have revised the text as per suggestions in abstract and methodology section in revised manuscript.

**Need to define what is meant by 'snake rescuers'.**

**Response:** 'Snake Rescuers' or 'Snake Catchers' or ‘ Snake Friends’ are the community volunteers who can handle live snakes. They voluntarily provide an important service to the community, by rescuing and relocating snakes that have been found near or even inside people's homes. There are detailed guidelines for Snake rescuers in Maharashtra State ( Source <https://kedarbhide.com/wp-content/uploads/2018/01/Guidelines-for-Snake-Rescue-and-Release-in-Maharashtra.pdf>). There are specific requirements to become snake rescuers. They should have sufficient training scientific knowledge about snake and experience of handling snakes of the area. They are involved regularly in public awareness about importance of snakes role in nature conservation. We have included the brief information about 'Snake Rescuers' in the revised manuscript page no. 10, line no, 216.

**Line 92: It is mentioned that snakebite is a forgotten issue. WHO has now a Global Snakebite Initiative in place and has published a strategy document.**

**Response:** We agree with the reviewer and we have replaced the earlier sentence with the following sentence ( Page no. 4, line nos. 79-81):

Snakebite envenoming has been reinstated by WHO as a high-priority neglected tropical disease (NTD) [1]. Recently, WHO launched its strategy for reducing the mortality and disability associated with snakebite envenomation to 50% by 2030 [2].

**108: What is meant by "....unreported mostly"? The two articles referenced are from Sri Lanka and India. No clear whether under reporting everywhere or in these two countries. What proportion of people used public healthcare services in these two countries or is this statement about all countries**

**Response:** We intended to share the information that snakebite cases could be underreported especially in rural areas. We have modified the sentence as follows (page no. 5, Line nos. 105-108 ) :

The morbidity and mortality of snakebites are usually underreported especially in rural areas as majority of the victims first report to traditional healers [16] and hence not all snakebite victims are treated in public health facilities[17][18].

Currently, there is no National data on proportion of people who used public healthcare services in India and Sri Lanka for snakebite treatment. Community-based surveys in Burdwan district in West Bengal, India showed higher annual mortality rates (16.4 deaths/100,000 population) [Hati AK et al 1992]. In the Sri Lanka study, 36.1% of snakebite deaths occurred outside the hospital, and another 26.4% of the victims who died reached hospital too late to receive any treatment due to poor transport and communication facilities, and a preference to seek traditional treatments for snakebite, which are more easily accessible for the communities.

**Overall, the Introduction section is focused on incidence of bites, deaths and use of public sector health services. The objective of the study is about awareness and perceptions. Therefore, it is important to introduce the concepts of 'awareness and perception' and its significance for prevention, first aid, early and effective treatment.**

**Response:** As suggested, we have added the following text in the introduction section of the revised manuscript (Page no. 6-7, Line nos. 124-152 ):

In India, snakebite is an occupational hazard affecting agricultural workers, farmers [26]. Working in fields, fetching drinking water, sleeping on floor, going to school or outdoor activities without footwear, defecation in open field or outdoor toilet are some of the activities associated with higher incidence of snakebites in rural areas [27,28]. Inappropriate perception, practice of unproven traditional methods and inadequate knowledge about snakes and snakebites in the vulnerable population may increase the snakebite mortality [29,30]. Most of these deaths are preventable, hence community awareness is crucial. Snakebite mortality is further increased by time lapsed after the snakebite, delay in ASV administration, inadequate administration of the initial dose of ASV, absence of simple methods of tracheal intubation and ventilation by a bag valve mask or artificial ventilator [28,31]. The community should be aware of the occupational risks and simple, cost effective preventive measures can prevent a snakebite. There is a need to increase community awareness on prevention of snakebites, bring behavioral changes regarding occupational risk and its reduction and empower the community on first aid skills and early transfer of snakebite patients to nearest health facility to avoid delay in ASV administration to improve the outcome.

Previously, we reported that there was no awareness and prior training on management of snakebites, no uniform protocol for treatment, use of intradermal ASV test in tribal region of Maharashtra [32,33]. Therefore, the present study was undertaken with following objectives : a) to understand awareness and knowledge about prevention, first aid practices and healthcare seeking behavior of the community for snakebite, b) to understand the awareness, knowledge and management practices for snakebite among the traditional faith healers, snake rescuers, frontline health workers, and Medical Officers c) assess ASV availability and utilization in public healthcare facilities and d) provide training to the healthcare workers in tribal region.

**166: June 2016 to October 2018 is mentioned as the study period. During that period, when exactly (which months/year) FGDs were conducted?**

**Response:** FGDs were conducted from July 2017 to December 2017. The information is mentioned in the revised manuscript, page no. 8, line no. 168.

**184: It is mentioned that the data about age was not collected. Young and old may have different perceptions, beliefs and knowledge. Similarly, educated and other may have different understanding of the issue. There is need to define in more detail who were the participant of these 18 FGDs i.e. what was the recruitment criteria? Did the authors attempted to include young, old, men, women, educated and those who have not attended the school etc.**

**Response :** We agree with reviewer that young and old may have different perceptions, beliefs and knowledge. Similarly, educated and other may have different understanding of the issue. Although, we have not collected the information on exact age of each participant attending the FGDs. The recruitment criteria was primarily focused on ensuring the representation of the diversity of the population in the study area. We conducted separate FGDs for men and women. We attempted to include older and younger participants in the community, community leaders and members, educated and uneducated participants.

**191: Need to inform about the role of the supervisor. Was the researcher present at the FGDs?**

**Response:** All FGDs were facilitated by supervisor. The facilitator is a social scientist from the research team and he was present at the FGD. Supervisor communicated with the peripheral health workers and community leaders for finalizing the date and time for FGD. The information about participation in FGD was given to the community through peripheral health workers and community leaders. Once the date was finalized, supervisor identified the venue for conducting the FGD in consultation with peripheral health workers and community leaders. He contacted the peripheral health workers and community leaders a day before the scheduled FGD for reminding them and re-confirming the participation of the invited participants from the community. He also ensured that a diverse group of participants are recruited for representing older and younger participants in the community, community leaders and members, educated and uneducated participants having an opportunity to participate in the discussion. On the day of FGD, supervisor carried the printed consent forms in vernacular language, audio recorder, stationary and ensured the necessary logistic arrangements for conducting the discussion. He was assisted by two social workers for writing the notes and recording the discussion. He introduced himself and his team of 2 MSWs. He briefly introduced the participants about purpose of the focus group and provided a copy of participant information sheet and informed consent form in vernacular language (Marathi). After obtaining the written informed consent, facilitator assigned the responsibility to two MSWs for taking notes, monitoring the audio recording and also checking the timer for keeping the track of time. He facilitated the discussion on perception, awareness and practices about snakebite in the community by encouraging participants to explore the discussion topics in depth and also giving the participants an opportunity to raise their own issues. He ensured that the handwritten notes and recordings are collected at the end of the FGD.

**192 How many Trained Medical Workers conducted 18 groups. One or 18? As that may have an impact on the data due to the potential variation between different FGD facilitators.**

**Response :** All 18 FGDs were carried out by the same facilitator with the help of two trained medical social workers to avoid any impact on the data due to the potential variation between different FGD facilitators. The facilitator is a social scientist from the research team.

**195: It is mentioned that "Once this discussion was over, the trained MSW introduced an FGD guide developed for the study. The total FGDs were divided into 3 themes and sub/minor themes. Questions focused on the general awareness ............". It seems that this was not a focus group discussion but a questionnaire administered to the individuals at the group. This needs to be explained, and the FGD Guide should be included in the in the supplementary material.**

**Response:** As suggested we have included FGD guide as a supplementary text S1. The method section is revised to explain clearly addressing the reviewers queries.

**202: The questionnaire was administered to various types of health care providers. Was there a different questionnaire for each type of healthcare providers as some are involved in managing snakebite patients while others may not?**

**Response:** The different questionnaires were used for different levels of the healthcare providers.

**211 Not clear what is meant by "...basic investigations". Need to inform what were those investigations.**

**Response :** By basic investigations we meant the bedside 20 minute whole blood clotting test (20 WBCT), Urine examination for albumin and blood by dipstick at primary health center level.

**223: It appears as if the survey was carried out just before the commencement of the training and then repeated immediately after the training. The results might reflect what was taught/discussed at the training as most will probably remember the concepts discussed for a short period of time immediately after a training but may not retain the knowledge if they are not routinely involved in the prevention and first aid community education or if they are not managing the patients on a regular basis. Additionally, this method and aspect of the research does not fit with the objective of the study. This is about the effectiveness of a training program.**

**Response :** We agree with the reviewer comment that the pre and post-test to assess the knowledge and management practices was carried out just before commencement of training and then repeated immediately after the training of Medical officers. Therefore, results of pre and post training survey might reflect what was taught/discussed at the training. However, these Medical Officers are working at different levels of public health care facilities (PHCs, RH and SDHs) in the Dahanu block and most of them are actively involved in the management of snakebite cases at their respective public health hospitals. Repeated training programs are conducted and Standard Treatment Guidelines (STD,2017) of Government of India is implemented in the study area. This aspect has been included as one of limitations of the study. Necessary changes are done in the revised manuscript, page no. 27, line nos. 596-602.

**257: Not clear what is meant by 'Circulating snakes'? Does it mean the snakes that are usually prevalent in the area and cause bites/envonoming?**

**Response :** Yes, circulating snakes mean the snakes that are usually prevalent in the area and cause bites/envenoming.

**Table 1. Did all participants agree on which of snakes were venomous or non-venomous? across all 18 groups? Did the participants discuss what is the general situation? There is some discrepancy between the information provided in the text and verbatim quotes (line 281-283) and the figure 1.**

**Response:** No not all participants agree on which of snakes were venomous or non-venomous across all 18 groups. Yes, the participants did discuss about the general situation. As mentioned in earlier response, we reemphasize that our aim is to share the information on community perception and knowledge about venomous and non-venomous snakes in the tribal block of Dahanu. Therefore, the information provided in Table 1 depicts the overall perception and knowledge about venomous and non-venomous circulating snakes in the Dahanu block and does not mean that all the participants agreed about the knowledge among the community at large. There is no discrepancy between information provided in the text and verbatim quotes and Table 1(we assume that reviewer wanted to mention about Table 1 instead of figure 1).

**289: It is mentioned that "Many of them preferred to visit nearby government healthcare facilities for snakebite treatment". Did FGD participants discuss why the rest of the community use/not use use the health care services? and what do they think what were the reasons?**

**Response:** Yes, the FGD participants discussed why the rest of the community do not use the public health care services in Dahanu block. Some of the participants narrated their past experiences and mentioned that they don’t prefer to visit government hospitals for snakebite treatment since they did not respond to emergency situations earlier, proper care is not given in government hospitals. Some of them responded that the community believes in the home remedy or traditional faith healers for snakebite treatment. The information is included in the revised manuscript, page no. 22, line nos. 488-491.

**287: Community awareness section is too short, considering that the main objective of the study is about awareness and perceptions. The discussion should have explored for information if there is change over time with regard to peoples awarenss, practices. Another area of expand is about peoples' perception about the snakes. For example is i mentioned that Cobra is considered as god. What are the community's beliefs about other snakes, but what about other snake? Did people have similar concepts about those snakes?**

**Response:** As suggested we have expanded the community awareness section and discussed the information on awareness and perception in details, Page nos. 20-23, line nos. 446-519.

**301. Did the group mentioned any other practices? Did the discussion took place about the whole community or whether the questions to the group participants were targeted at the practices of the respondent”?**

**Response :** The group did not mention any other practices**.** The questions were targeted at the practices of the respondents.

**307: The quote (about bites to the animals) presented here does not belong to this heading.**

**Response :** We agree with the comment and quote (about bites to the animals) is deleted in the revised manuscript, page no. 14, line nos. 316-318.

**325: About the face to face interviews with health care providers, what was the sample size?**

**Response:** The research team visited health care facilities (Sub centers, PHCs, RH and SDHs) in Dahanu block to conduct the face to face interviews. Based on the availability and willingness to participate in the study, the health care workers ( ASHA, MPW, ANM, Nurse) were invited to participate. We were able to conduct the face to face interviews of 96 health care providers in study area. The information is included in revised manuscript page no. 15, line no. 337.

**333 Were the respondent shown photos or venomous and non-venomous for them to point out which**

**Response :** No, the respondents were not shown photos or venomous and non-venomous snakes.

**Page 17: The question “Are you aware of snakebite cases in your area?” - Needed to ask with a reference to a time frame**

**Response :** We agree with the suggestion. We understand that this question should have been asked with a reference to a time frame.

**344: Among the 15 nurses who said that they have seen 25-50 cases in 6 months, did they work at the same healthcare facility?**

**Response:** Yes, they work at the same healthcare facility.

**461: It is mentioned that "the belief that after the snakebite, if the victim cannot walk even 5 steps considered as a venomous snakebite". Is this belief held by one who mentioned it or if many or all FGD participants agreed with it, across all FGDs?**

**Response :** This belief was mentioned by only one female participant in FGD conducted at PHC Aina (FGD ID 08).

**505 what Is meant by Ayurveda discipline' ?.**

**Response :** Ayurveda is one of the most ancient systems of medicine serving people in the Indian subcontinent. Bachelor of Ayurvedic Medicine and Surgery (B.A.M.S.) is a professional degree in Ayurveda offered in India, Nepal, Bangladesh, and other South Asian countries.

**Response to comments PNTD-D-20-00439R1**

**Perceptions, awareness on snakebite envenomation among the tribal community and health care providers of Dahanu block, Palghar district in Maharashtra, India**

**Comment 1 : The writing of objectives should be improved. Suggest replacing the word ‘understand’ with explore/ describe.**

**Response:** We have replaced the word ‘understand’ with explore. Necessary changes are made in the revised manuscript, page no. 2, line no.29, page no. 7, line no. 146, 148.

**Comment 2 : The training provided to health staff should not be presented as an objective of this paper. As previous reviewers have suggested, the attempt to include all the components of the study in one paper has reduced the focus of this paper. This paper should focus on previously unexplored aspect on perception and knowledge on snakebite in the local community and health staff.**

**Response:** We respect the in-depth review of our manuscript. However, we disagree with the point that the training provided to health staff should not be presented as an objective of this paper. The justification for inclusion of the training of health staff in the manuscript is as follows:

The draft of the Standard Treatment Guidelines (STGs) of Government of India for management of snakebite was released in 2016. Prior to our study, there was no evidence on feasibility of implementation of the STGs in India. It was very crucial to generate evidence on feasibility of implementation of STGs. Therefore, one of the objectives of our study was to implement the STGs for capacity building on prevention, diagnosis and management of snakebite. Intense training was provided to the Medical Officers on prevention, diagnosis and management of snakebite as per the STGs. Pre and post training test was conducted using a self-administered questionnaire for assessing the knowledge prior to training and improvement after training. Repeated training programs were conducted for the Medical Officers to assess knowledge retention and improvement in management practices. Since snakebite is a major burden in the study area, capacity building of healthcare providers was a major part of our study. Therefore, through the present manuscript we are giving complete information related to the work carried out in the tribal community that resulted into a model. We strongly feel that by combing all components of the study in one paper, the readers will get the complete information in one paper. Therefore, we included the information about all activities in the same manuscript.

**Comment 3: The authors should get assistance from a researcher experienced in qualitative research to present the results of the qualitative component of this study in a meaningful way. The presentation of qualitative data is weak and superficial. There should be deeper exploration of findings of the qualitative study in both the Results and Discussion sections.**

**Response:** We respect the view of the reviewer. We are humbly informing that one of the joint first authors (DA) is a qualified social scientist and experienced researcher in qualitative research. Dr Himmatrao Bawaskar, one of the global authorities on snakebite research is one of the coauthors and he was involved in implementation of study, data analysis, interpretation and drafting the manuscript. Additionally, we also consulted the experienced scientist in qualitative research to evaluate our manuscript independently. The manuscript especially qualitative component of the study was critically reviewed independently by a senior researcher having experience in qualitative research ( YK). DA is joint first author and YK is acknowledged for his critical review and inputs for improving the manuscript. We, therefore emphasize that the revised manuscript submitted to PLOS NTD based on comments of reviewers has deeper exploration of findings of the qualitative study in both the Results and Discussion sections. Hence, we do not agree with rewriting the qualitative part of the manuscript.

**Comment 4 : There is very little published literature on the role of traditional healers and snake rescuers in South Asia. This aspect of the paper should be given more focus when re-writing this paper.**

**Response:** We agree with the reviewer that there is very little published literature on the role of traditional healers and snake rescuers in South Asia. However, in the revised manuscript submitted to PLOS NTD we had already expanded this aspect and given more focus based on earlier reviewer’s comments. The information on role of traditional healers and snake rescuers is described in detail in the revised manuscript [Page no. 5, line 107-108, Page no. 6, line no. 127-129, Page no. 7, line no. 148-149,Page no. 10, line no. 216-218, 226-227, Page no. 12, line 267-268, Page no. 14, line no. 323-326, Page no.15.16, 334-375, Page no. 23, Line no. 476-519, Page no. 23, line no. 504-519, Page no. 26, line no. 589-591, Page no. 27, line no. 596-597].

**Comment 5 : The relatively large sample size for each focus group may have affected the quality of data adversely, as many participants could be unwilling to provide information freely in a large group. This needs to be discussed under limitations.**

**Response:** We thank reviewer for the suggestion. We have added required text in the limitations. Page 27, line 593-596

**Minor comments**

1. **The writing style should be improved with more attention to grammar.**
2. **Please re-organise the content to bring out the importance of findings which are unique to this paper.**

**Response:** We have done the corrections wherever needed.

In the revised manuscript, we have done all the corrections as per the comments. However, for some of the comments we have provided our explanations and we hope that the manuscript will now be accepted without any further changes.

We look forward positive consideration of our manuscript for publication in PLOS One.

Thanking you

Sincerely,

Dr Rahul Gajbhiye
