# Supplementary material for: Perceptions, awareness on snakebite envenoming among the tribal community and health care providers of Dahanu block, Palghar District in Maharashtra, India
Source: PLoS One. 2021 Aug 5;16(8):e0255657. doi: 10.1371/journal.pone.0255657 (PMC8341635; doi:10.1371/journal.pone.0255657)
Supplement: Attachment — Submitted filename: Comments to the authors.docx [file pone.0255657.s008.docx]

**Comments to the authors**

**Abstract**

- Line 35 – In-depth-interviews are to be conducted using interview guide, not using pre-tested questionnaire..

**Methodology & Results**

- As the study is of qualitative nature, authors need to refer to COREQ checklist (<http://cdn.elsevier.com/promis_misc/ISSM_COREQ_Checklist.pdf>) and report the data analysis and study results.
