# Supplementary material for: Perceptions, awareness on snakebite envenoming among the tribal community and health care providers of Dahanu block, Palghar District in Maharashtra, India
Source: PLoS One. 2021 Aug 5;16(8):e0255657. doi: 10.1371/journal.pone.0255657 (PMC8341635; doi:10.1371/journal.pone.0255657)
Supplement: Attachment — Submitted filename: Response to Reviewers.docx [file pone.0255657.s009.docx]

Dear PLOS ONE Editorial Staff,

Subject: Point by point response to reviewers and editors comments for the manuscript “Perceptions, awareness on snakebite envenomation among the tribal community and health care providers of Dahanu block, Palghar district in Maharashtra, India”

Please find below our point-by-point response to comments of the academic editor and reviewers. Many thanks for your efforts on this manuscript.

**Editors Comments**

**1.** Please ensure that your manuscript meets PLOS ONE's style requirements, including those for file naming. The PLOS ONE style templates can be found at

https://journals.plos.org/plosone/s/file?id=wjVg/PLOSOne_formatting_sample_main_body.pdf and

https://journals.plos.org/plosone/s/file?id=ba62/PLOSOne_formatting_sample_title_authors_affiliations.pdf

Resposne: Thank you. We have tried our level best to format our manuscript according to the PLOS ONE’s style requirements. However, if there are any omissions, please point them out. We will continue to make corrections.

**2.** Please include your tables as part of your main manuscript and remove the individual files. Please note that supplementary tables should remain uploaded as separate "supporting information" files.

Resposne: Tables are included as part of main manuscript and supplementary tables are submitted as supporting information.

**3.** We suggest you thoroughly copyedit your manuscript for language usage, spelling, and grammar. If you do not know anyone who can help you do this, you may wish to consider employing a professional scientific editing service.  

Whilst you may use any professional scientific editing service of your choice, PLOS has partnered with both American Journal Experts (AJE) and Editage to provide discounted services to PLOS authors. Both organizations have experience helping authors meet PLOS guidelines and can provide language editing, translation, manuscript formatting, and figure formatting to ensure your manuscript meets our submission guidelines. To take advantage of our partnership with AJE, visit the AJE website ([http://learn.aje.com/plos/](about:blank)) for a 15% discount off AJE services. To take advantage of our partnership with Editage, visit the Editage website (www.editage.com) and enter referral code PLOSEDIT for a 15% discount off Editage services.  If the PLOS editorial team finds any language issues in text that either AJE or Editage has edited, the service provider will re-edit the text for free.

Upon resubmission, please provide the following:

- The name of the colleague or the details of the professional service that edited your manuscript
- A copy of your manuscript showing your changes by either highlighting them or using track changes (uploaded as a *supporting information* file)
- A clean copy of the edited manuscript (uploaded as the new *manuscript* file)

Resposne: The manuscript has been edited for language usage, spelling, and grammar as per the recommendations.

**4.** In the Methods, please discuss whether and how the questionnaire was validated and/or pre-tested. If these did not occur, please provide the rationale for not doing so.

**Resposne:** Thank you for the suggestion. We have added additional information about the questionnaire including validation and pre-testing. This information has been added to the ‘Data collection Tools’ sub-section of the “methods” section **(**Page No. 08; Line no. 170).

**5.** Thank you for stating the following in the Funding Section of your manuscript:

"RG is an awardee of the DBT
 Wellcome India alliance clinical and public health intermediate fellowship (Grant no.
IA/CPHI/18/1/503933)."

We note that you have provided funding information that is not currently declared in your Funding Statement. However, funding information should not appear in the Acknowledgments section or other areas of your manuscript. We will only publish funding information present in the Funding Statement section of the online submission form.

Please remove any funding-related text from the manuscript and let us know how you would like to update your Funding Statement. Currently, your Funding Statement reads as follows:

"Initials of the authors who received Funding : RG, SM

Grant numbers awarded :

Tribal Health Research Forum, Indian Council of Medical Research and Department of Health Research, Ministry of Health and Family Welfare, Government of India for financial assistance (NIRRH/MS/RA/886/03/2020)

The full name of each funder
URL of each funder website

www.icmr.nic.in

Did the sponsors or funders play any role in the study design, data collection and analysis, decision to publish, or preparation of the manuscript?

NO"

Please include your amended statements within your cover letter; we will change the online submission form on your behalf.

**Resposne:** Thanks for pointing out the problem.

We have deleted the statement RG is an awardee of the DBT
 Wellcome India alliance clinical and public health intermediate fellowship (Grant no.
IA/CPHI/18/1/503933) from the acknowledgement section.

6. We note that Figure 1 in your submission contain map images which may be copyrighted. All PLOS content is published under the Creative Commons Attribution License (CC BY 4.0), which means that the manuscript, images, and Supporting Information files will be freely available online, and any third party is permitted to access, download, copy, distribute, and use these materials in any way, even commercially, with proper attribution. For these reasons, we cannot publish previously copyrighted maps or satellite images created using proprietary data, such as Google software (Google Maps, Street View, and Earth). For more information, see our copyright guidelines: http://journals.plos.org/plosone/s/licenses-and-copyright.

We require you to either (1) present written permission from the copyright holder to publish these figures specifically under the CC BY 4.0 license, or (2) remove the figures from your submission:

6.1.    You may seek permission from the original copyright holder of Figure 1 to publish the content specifically under the CC BY 4.0 license.

We recommend that you contact the original copyright holder with the Content Permission Form (http://journals.plos.org/plosone/s/file?id=7c09/content-permission-form.pdf) and the following text:

“I request permission for the open-access journal PLOS ONE to publish XXX under the Creative Commons Attribution License (CCAL) CC BY 4.0 (http://creativecommons.org/licenses/by/4.0/). Please be aware that this license allows unrestricted use and distribution, even commercially, by third parties. Please reply and provide explicit written permission to publish XXX under a CC BY license and complete the attached form.”

Please upload the completed Content Permission Form or other proof of granted permissions as an "Other" file with your submission.

In the figure caption of the copyrighted figure, please include the following text: “Reprinted from [ref] under a CC BY license, with permission from [name of publisher], original copyright [original copyright year].”

6.2.    If you are unable to obtain permission from the original copyright holder to publish these figures under the CC BY 4.0 license or if the copyright holder’s requirements are incompatible with the CC BY 4.0 license, please either i) remove the figure or ii) supply a replacement figure that complies with the CC BY 4.0 license. Please check copyright information on all replacement figures and update the figure caption with source information. If applicable, please specify in the figure caption text when a figure is similar but not identical to the original image and is therefore for illustrative purposes only.

The following resources for replacing copyrighted map figures may be helpful:

USGS National Map Viewer (public domain): http://viewer.nationalmap.gov/viewer/

The Gateway to Astronaut Photography of Earth (public domain): http://eol.jsc.nasa.gov/sseop/clickmap/

Maps at the CIA (public domain): https://www.cia.gov/library/publications/the-world-factbook/index.html and https://www.cia.gov/library/publications/cia-maps-publications/index.html

NASA Earth Observatory (public domain): http://earthobservatory.nasa.gov/

Landsat: http://landsat.visibleearth.nasa.gov/

USGS EROS (Earth Resources Observatory and Science (EROS) Center) (public domain): http://eros.usgs.gov/#

Natural Earth (public domain): <http://www.naturalearthdata.com/>

**Response:** Thanks for your detailed guidance. We requested the copyright agency for the same, and we received “Email consent approval” to use the map (copy attached as Other File).

7. We note that Figures SF2 and SF3 include images of participants in the study.

As per the PLOS ONE policy ([http://journals.plos.org/plosone/s/submission-guidelines#loc-human-subjects-research](about:blank#loc-human-subjects-research)) on papers that include identifying, or potentially identifying, information, the individual(s) or parent(s)/guardian(s) must be informed of the terms of the PLOS open-access (CC-BY) license and provide specific permission for publication of these details under the terms of this license. Please download the [Consent Form for Publication in a PLOS Journal](about:blank) ([http://journals.plos.org/plosone/s/file?id=8ce6/plos-consent-form-english.pdf](about:blank)). The signed consent form should not be submitted with the manuscript, but should be securely filed in the individual's case notes. Please amend the methods section and ethics statement of the manuscript to explicitly state that the patient/participant has provided consent for publication: “The individual in this manuscript has given written informed consent (as outlined in PLOS consent form) to publish these case details”.

If you are unable to obtain consent from the subject of the photograph, you will need to remove the figure and any other textual identifying information or case descriptions for this individual.

**Response:** Thank you for guidance. We have removed the SF2 and SF3.

**Resposne to Reviewers comments**

**Reviewer #1:**

The research question is definitely of interest to readers in India and other countries in the tropics where snakebite is well and truly a occupational hazard. To the best of my knowledge a study along similar lines has not been carried out in India and the results of the study would help policy makers in bringing in changes to plug the gaps and deficiencies identified. The research question interesting and the methodology fairly well charted.
The deficiency really is in the presentation of the findings and results.

The abstract and introduction need to be written better and be representative of the actual research question. The introduction seems scattered and not to the point, going from numbers of snakebite to chronic sequelae which also includes death, venom constituents and effect to ASV. Restructuring the introduction and giving it a form should help make it read better. Would suggest adding some details on the tribal community and geography as well. The research question here is perceptions and awareness on snakebite envenomation, of the 202 respondents in the community it has not been explicitly stated whether all were tribals. The researchers could also specify whether the different tribes had different occupations vis a vis hunter, gatherer, farmer etc and their educational status.

**Response:** We thank the reviewer for suggestions to improve the strength of our manuscript. As per the reviewer’s recommendation, we have revised the abstract, introduction and also added additional information on details on the tribal community and geography. Additionally, the information different tribes and their occupations in study area is also added in the introduction section (Introduction - Page no. 06, line no. 124 -146).

We greatly appreciate the reviewer’s critical comments and recommendations.

Line 62; higher than what, as only 13 states were assessed as a part of the million death study.

**Response:** The sentence is revised as follows:

An earlier study conducted in India demonstrated the highest snakebite mortality rates per 100,000 with an average prevalence of 4.5 and varied between states from 3.0 (Maharashtra) to 6.2 (Andhra Pradesh), compared to (1.8) the rest of country”. (Manuscript page no. 03, line no 63-66).

Line 82 unidentified- unknown

**Response:**

As suggested, we have replaced the word unidentified with Unknown. (Manuscript page no. 04, line no 83).

Lines 84,85 - it would be interpreted that death is a long term complication

Response:

We agree with the reviewer's suggestion. We have removed the word “Death”. (Manuscript page no. 04, line no 87)

Lines 94,95- Relevance of skin hypersensitivity in the context of the article

Response: We agree with the reviewer comment. The text on skin hypersensitivity is deleted.

103-108 against the flow

Response: Thank you. The text (103-108) is deleted.

Line 125 drinking water (potable water), would also imply sleeping is an activity?

Response: We agree with the reviewer's suggestion, changed the word drinking water with “Potable water. Removed the sleeping from activity. (Manuscript page no. 05, line no 103 -104)

Lines 139-141 – 4.5 % CFR included non venomous too (76% venomous and 24% non venomous)

Response: The annual incidence of snakebite was 36 per 100,000 populations in Dahanu block. The total venomous bites were 110 of which five have died; So CFR is 4.5%. This does not include non-venomous bites. (Manuscript page no. 06, line no 118).

Line 165 qualitative study, qualitative cannot be considered a study design, the study is cross sectional

Response: We thank the reviewer for suggestion on study design. As recommended, the study design is changed to cross sectional study”. (Manuscript page no.07, line no 149).

Line 185 convenience sampling ? how representative would it be of the population studied

**Response:** The recruitment criteria was primarily focused on ensuring the representation of the diversity of the population in the study area. We conducted separate FGDs for men and women. We attempted to include older and younger participants in the community, community leaders and members, educated and uneducated participants. Majority of the FGD participants were representative of the tribal and non-tribal population in study area. This has been explained under methods section.

Line 207 it could be that they are unaware of their age

**Response:** Yes we agree. It may be possible that the participants were unaware of their age. We have included this sentence in the revised manuscript (Page no. 10 , line no. 208-209).

Line 345/443 peripheral health care workers, would be best sticking to frontline health workers

Response: We thank for the suggestion. Peripheral health care workers is replaced by frontline health workers. Recommended changes are done throughout the Manuscript.

Line 395 how were the 18 out of 65 SC decided upon, what criteria was used to pick the same

Response: The 18 out of 65 sub centers were selected by random selection method ( Page no.11, Line no. 239).

Line 457 / 58 not clear what he intends to say is it that non venomous do not bite multiple times

Response: We have deleted the sentence line 457/58.

The training for medical personnel seems not to have made much of a difference in improving knowledge of treating doctors

Response: We agree that the training of medical officers did not made much of a difference in improving of treating doctors. The possible reasons could be the educational background of the MOs who attended the training. Seventy two percent (18 out of 25) of the MOs were from Ayurveda discipline (non MBBS doctors) and only seven MOs were having qualifications of MBBS. Ayurveda is one of the most ancient systems of medicine serving people in the Indian subcontinent. Bachelor of Ayurvedic Medicine and Surgery (B.A.M.S.) is a professional degree in Ayurveda. We therefore conducted repeat training programs in the study area to improve the knowledge of MOs.

Was there an incentive given to the community responders, was it a convenience sampling and if so would it bias the results and could the result be considered as representative of the study population

Response: No incentive was given to the community responders and the participation in FGD was voluntary. Convenience sampling method was used for selection of participants for focus group discussions. We included participants of both genders reflecting a wide range of ages, community leaders and members, older and younger participants representing the tribal and non-tribal population to avoid any bias.

The most important part of the result would be the perception and understanding of the subject of venomous snakebite among the community members presumably tribals, this for me, the qualitative study results are not clear. Themes and sub themes have been mentioned but only a few have been represented. If a table giving percentages of themes and subthemes were displayed the qualitative aspect would be much clearer. The qualitative analysis needs better representation both by way of themes and subthemes and its relevance to the community studied.
I take this opportunity to wish the researchers the best and hope they continue their research in this much neglected field.

Response: We truly appreciate the reviewer’s in depth review of our manuscript and bringing out the shortcomings of our manuscript. This has helped us in improving the manuscript and presentation of the results of FGDs with more clarity. As per the recommendation, a table is prepared giving percentages of the subthemes. We take this opportunity to thank the reviewer for appreciation and motivating us to continue our research in this much neglected field.

| **Major themes and Sub- themes of the study** | **Frequency** | **Percentage** |
| --- | --- | --- |
| **Major theme 1: Awareness and Knowledge about snakes** | | |
| Names of venomous and non-venomous snakes | 120 | 59.4% |
| Hiding places of snakes | 106 | 52.5% |
| Identify venomous snakes | 66 | 32.6 % |
| Season and timing of snakebites | 32 | 15.8% |
| **Major theme 2: Perception and health seeking behavior for snakebite** | | |
| Beliefs related to snakebites | 37 | 18.3% |
| Treatment at public health facility | 67 | 33% |
| Treatment from traditional faith healers | 24 | 11.8 % |
| Application of herbal products or home remedies | 25 | 12.3% |
| **Major theme 3: Knowledge on prevention, first aid and treatment for snakebite** | | |
| Preventive measures awareness | 35 | 17.3% |
| Use of tourniquet/ bandage | 29 | 14.3% |
| Availability of snakebite treatment at public hospitals | 17 | 8.4% |
| Transport to nearest public health facility | 18 | 8.9% |

Reviewer #2: Abstract
Line 35 – In-depth-interviews are to be conducted using interview guide, not using pre-tested questionnaire.
Response: We thank the reviewer for pointing out the mistake. We apologize for the inconvenience. We have made necessary changes in the manuscript.

Methodology & Results
As the study is of qualitative nature, authors need to refer to COREQ checklist (<http://cdn.elsevier.com/promis_misc/ISSM_COREQ_Checklist.pdf>) and report the data analysis and study results.

Response: Thank you for the recommendation of COREQ checklist. As per the recommendation, COREQ check list is included in the manuscript under method section (S1 text). (Manuscript Page no 07. line no. 158)

**Reviewer #3:**

1. The area of this research is important for India where more than 50% of global snakebite deaths are happening there. A recent study published in E-life by Million Death Study collaborates finds 58k deaths and 1.1 to 1.7 million bites annually in India and one million deaths were in last 20 years. I learnt from a snakebite advocacy group meeting at ICMR, a substantial amount of expired unused anti venom vials throwing into trash without use in every year while many thousands die without treatments. This is a problem of either people not aware of (or trust) anti venom treatment in hospitals or physicians were not trained/confident enough in clinical management of snakebite cases or both.

Response: We thank the reviewer for acknowledging the importance of snakebite research for India.

2. According to the authors, the objective of this study is to test the awareness about prevention strategies, knowledge of first aids and treatment options among community; Snakebite patient management among snake handlers, healers, healthcare workers and medical personnel. Another set objective is to train the healthcare workers, a significant part of the manuscript allocated for that purpose. I am not sure the latter is a research?

Response: As recommended, we have deleted the major text related to training of healthcare workers.

3. I have some doubt of technical aspect of the methods. As describe in the manuscript this study is a qualitative research based on focus group discussions. At the same time, they use pretested questionnaires for data collection. To my knowledge, data were collected using recorded narratives in qualitative research instead use questionnaires. Information of these data are analyzed using standard software like Nvivo. It is difficult for me to comment anything about the method because this method is a mix of quantitative survey methods and qualitative research, which I am not familiar.

Response: We apologize for not clearly presenting the qualitative results. As recommended by reviewer, the methodology and results are revised and reported as per COREQ checklist (Page no. 7, Line no 158). We did not use the software Nvivo and manual coding was done.

4. Commenting of statistical analysis and presentation, tables and in figures are very poorly presented. In particularly figures are powerful tools to exhibit study outcomes. It is difficult to grasp the messages going to reflect from figures and they should need significant improvements to bring into the journal standard.

Response: As recommended we have substantially edited the manuscript, tables, and figures for improved presentation as per the journal standard.

5. Full of many unwanted stories, inclusion of unrelated stuffs, repeating the same information in many places, confusing non-standard English wordings and confusing sentences etc. are in everywhere of this manuscript and very hard to follow for me to read and understand about 60 pages of the manuscript.

Response: We apologize for the inconvenience caused to the reviewer. As recommended, we have revised the manuscript in light of the reviewer’s comments.

6. Research reporting also seen some professional bias. Researchers as the allopathic medical personnel ask the traditional healers to disclose their treatments at focus group meetings in front of others. Researchers complaining they rejected to explain their treatment methods to them. I think it is not relevant for this study and these researchers don’t have a common sense about other professions.

Response: We did not conduct the FGD with traditional healers. We conducted interview of traditional healers individually and not in group. The reasons for not disclosing the treatment given by Traditional healers was explained in the manuscript.

7. I am sorry all my comments are negative here. It does not mean this manuscript does not have anything good. I didn’t spend my time to go too much deep commenting for improving the manuscript because so many technical flaws are already there.

Response: We sincerely thank the reviewer for accepting the fact that our manuscript has merit. As per the recommendations, we have revised the manuscript substantially incorporating the suggestions of all the reviewers and editor.
