# Supplementary material for: Perceptions, awareness on snakebite envenoming among the tribal community and health care providers of Dahanu block, Palghar District in Maharashtra, India
Source: PLoS One. 2021 Aug 5;16(8):e0255657. doi: 10.1371/journal.pone.0255657 (PMC8341635; doi:10.1371/journal.pone.0255657)
Supplement: Attachment — Submitted filename: To.docx [file pone.0255657.s010.docx]

To,

The Editor

PLOS ONE

Subject: Review of the manuscript titled **"Perceptions, awareness on snakebite envenomation among the tribal community and health care providers of Dahanu block, Palghar district in Maharashtra, India"**

The revised article makes for a better read and the suggestions of the three reviewers have been incorporated. The scientific/generic names of snakes have to be corrected. Echis sp not mentioned, as to whether carinatus / sochureki etc.

Wish the authors the best. I have no hesitation in recommending the manuscript for publication.

Thanks for having given me the opportunity to review this manuscript

Regards

Jaideep

Dr Jaideep C Menon

Cardiologist, Amrita Institute of Medical Sciences

Kochi
