# Supplementary material for: Perceptions, awareness on snakebite envenoming among the tribal community and health care providers of Dahanu block, Palghar District in Maharashtra, India
Source: PLoS One. 2021 Aug 5;16(8):e0255657. doi: 10.1371/journal.pone.0255657 (PMC8341635; doi:10.1371/journal.pone.0255657)
Supplement: Attachment — Submitted filename: Revewer report WS (post response).docx [file pone.0255657.s011.docx]

**Reviewer #3:**

1. The area of this research is important for India where more than 50% of global snakebite

deaths are happening there. A recent study published in E-life by Million Death Study

collaborates finds 58k deaths and 1.1 to 1.7 million bites annually in India and one million deaths

were in last 20 years. I learnt from a snakebite advocacy group meeting at ICMR, a substantial

amount of expired unused anti venom vials throwing into trash without use in every year while

many thousands die without treatments. This is a problem of either people not aware of (or trust)

anti-venom treatment in hospitals or physicians were not trained/confident enough in clinical

management of snakebite cases or both.

Response: We thank the reviewer for acknowledging the importance of snakebite research for

India.

*# Thanks.*

2. According to the authors, the objective of this study is to test the awareness about prevention

strategies, knowledge of first aids and treatment options among community; Snakebite patient

management among snake handlers, healers, healthcare workers and medical personnel. Another

set objective is to train the healthcare workers, a significant part of the manuscript allocated for

that purpose. I am not sure the latter is a research?

Response: As recommended, we have deleted the major text related to training of healthcare

workers.

*# Glad to see many improvements in the Introduction text. I like the new text from line 184 to 208 and is appropriate.*

3. I have some doubt of technical aspect of the methods. As describe in the manuscript this study

is a qualitative research based on focus group discussions. At the same time, they use pretested

questionnaires for data collection. To my knowledge, data were collected using recorded

narratives in qualitative research instead use questionnaires. Information of these data are

analyzed using standard software like Nvivo. It is difficult for me to comment anything about the

method because this method is a mix of quantitative survey methods and qualitative research,

which I am not familiar.

Response: We apologize for not clearly presenting the qualitative results. As recommended by

reviewer, the methodology and results are revised and reported as per COREQ checklist (Page

no. 7, Line no 158). We did not use the software Nvivo and manual coding was done.

*# Thanks for improvements in research method. It is now somewhat more comprehensible to me. Since my knowledge in qualitative research is limited, I leave it to other reviewers and the editor to take care.*

4. Commenting of statistical analysis and presentation, tables and in figures are very poorly

presented. In particularly figures are powerful tools to exhibit study outcomes. It is difficult to

grasp the messages going to reflect from figures and they should need significant improvements

to bring into the journal standard.

Response: As recommended we have substantially edited the manuscript, tables, and figures for

improved presentation as per the journal standard.

*# I am OK with the revision*

5. Full of many unwanted stories, inclusion of unrelated stuffs, repeating the same information in

many places, confusing non-standard English wordings and confusing sentences etc. are in

everywhere of this manuscript and very hard to follow for me to read and understand about 60

pages of the manuscript.

Response: We apologize for the inconvenience caused to the reviewer. As recommended, we

have revised the manuscript in light of the reviewer’s comments.

*# A good progress can be seen in revised manuscript. I think remaining repeating information and consistency of the text in the manuscript can take care by the editor with the help of the authors at the editorial process.*

6. Research reporting also seen some professional bias. Researchers as the allopathic medical

personnel ask the traditional healers to disclose their treatments at focus group meetings in front

of others. Researchers complaining they rejected to explain their treatment methods to them. I

think it is not relevant for this study and these researchers don’t have a common sense about

other professions.

Response: We did not conduct the FGD with traditional healers. We conducted interview of

traditional healers individually and not in group. The reasons for not disclosing the treatment

given by Traditional healers was explained in the manuscript.

*# I am OK with the revision of the text.*

7. I am sorry all my comments are negative here. It does not mean this manuscript does not have

anything good. I didn’t spend my time to go too much deep commenting for improving the

manuscript because so many technical flaws are already there.

Response: We sincerely thank the reviewer for accepting the fact that our manuscript has merit.

As per the recommendations, we have revised the manuscript substantially incorporating the

suggestions of all the reviewers and editor.

*# Thanks.*

\# Additional comments:

Throughout this manuscript uses the words ‘envenomation’ and ‘envenoming’ interchangeably. It looks authors have not given much attention to the difference between these 2 words. I think if the bite is an accident, then the best word would be the envenoming that WHO uses <https://www.who.int/snakebites/disease/en/>. Also I see another word “anti-snake venom”. The correct word should be the “anti-venom”. Refer same WHO and recent leading research articles on Snakebites.
