# Supplementary material for: Perceptions, awareness on snakebite envenoming among the tribal community and health care providers of Dahanu block, Palghar District in Maharashtra, India
Source: PLoS One. 2021 Aug 5;16(8):e0255657. doi: 10.1371/journal.pone.0255657 (PMC8341635; doi:10.1371/journal.pone.0255657)
Supplement: Attachment — Submitted filename: Response to Reviewers Comments .docx [file pone.0255657.s012.docx]

**RESPONSE TO REVIEWERS COMMENTS**

Dear PLOS ONE Editorial Staff,

Many thanks for your efforts on this manuscript entitled “Perceptions, awareness on snakebite envenoming among the tribal community and health care providers of Dahanu block, Palghar district in Maharashtra, India”.

Our point-by-point response to reviewer's and editor's comments are as follows.

**Journal Requirements:**

Please review your reference list to ensure that it is complete and correct. If you have cited papers that have been retracted, please include the rationale for doing so in the manuscript text, or remove these references and replace them with relevant current references. Any changes to the reference list should be mentioned in the rebuttal letter that accompanies your revised manuscript. If you need to cite a retracted article, indicate the article’s retracted status in the References list and also include a citation and full reference for the retraction notice.

Response: As suggested we have reviewed all the references quoted in the revised manuscript. We confirm that there are no retracted references cited in the manuscript.

**Academic editors comments**

1. -Few errors of English grammar and use persist in the revised manuscript. Copy-edit the manuscript before proceeding with the next submission.

Response: Thanks for pointing out the errors of English grammer. We have edited the manuscript and tried our best to rectify the grammatical errors in the revised manuscript.

2. - Avoid abuse of capital letters throughout the manuscript (examples: line 58 - replace ''Health care workers'' with ''health care workers''; line 50: replace ''Medical Officers'' with ''medical officers'')

Response: As suggested, we have done the changes throughout the revised manuscript.

3. - Abstract: ASHA? ANM? MPW?

Response: As suggested we have included full forms of ASHA, ANM and MPW in the abstract section (line no: 50 & 51) and deleted in the main text (Line no: 232 & 233).

4.- Line 113-Introduction: ASV - Provide the full form in the first place of use of the abbreviation in the main text.

Response: As recommended by reviewer 3, we have omitted the use of word anti-snake-venom (ASV) which is now replaced by “anti-venom” in the entire revised manuscript.

5. -Line 146-Introduction: HCW - Provide the full form in the first place of use of the abbreviation in the main text. I am aware that the abbreviation/full-form is already being considered in the 'abstract' section.

Response: . As suggested, changes have been done in the revised manuscript.

6. - Line 152- Methods: ICU & OPD - Provide the full forms.

Response: ICU & OPD full forms are added in the revised manuscript. Line No: 157,158

7.- Line 170 - Methods: PHC?

Response: PHC full form is added in the revised manuscript. Line No: 177 .

8- Line 171 - Methods: MSW?

Response: MSW full form is added in the revised manuscript. Line No: 178.

9.- Methods-Recruitment, Sampling & Data Collection: The use of the abbreviation DA in the text apparently seems to be confusing for a term rather than the initials of a co-author. Rewrite to avoid confusion as there are other abbreviations like FGD and HCW used in the same paragraph.

Response: Abbrivated authors names have been deleted in revised manuscript. Page No: 08 to10.

10.- Line 280 - Results: Would you prefer to substitute ''circulating snakes'' with a better phrase?

Response: Accepted the suggestion, ''circulating snakes'' phrase is replaced with “commonly found snakes in the area”. Line No: 291. The changes are done throughout the manuscript.

11. - Lines 306-312: The scientific name of Indian cobra is mentioned, but not that of the common krait, Russell's viper and saw scaled viper. All scientific names are mentioned along side the corresponding common names in Table 1. Therefore, for the sake of uniformity, avoid using the term Naja naja in this paragraph.

Response: Accepted the suggestion; the scientific name of Indian cobra is removed in the revised manuscript. Line no: 317.

12.- Let the ''conclusions'' drawn be based on the data/observations/results of the present study. Provide separate paragraphs on ''recommendations/future directions'' and ''implications of the present study''.

Response: As per the suggestions, we have revised the conclusion section. Also added separate paragraphs on recommendations and implications of the present study. Page no: 28, 29 line no: 593 & 607.

13. Address the minor revisions recommended by the reviewer(s).

Response : We have addressed the minor revisions recommended by the reviewers.

**Reviewer #1**

The comments of the three reviewers have been taken into account by the authors and necessary revisions made. The manuscript makes for a much better read now and would be of interest to readers. The scientific / generic names of the snakes to be written correctly separating genus / species. Mention to be made of which echis species- carinatus or sochureki.

Wish the authors the best and I would recommend the article for publication, which I am sure would be of interest to a lot of readers.

Response: We sincerely thank the reviewer for critical comemnts and suggestions. Genus and species names of snakes have been corrected. The name of echis species i.e *Echis carinatus* is added in the revised manuscript. Table 1, page no: 15; Line no. 305.

**Reviewer # 3**

Glad to see many improvements in the Introduction text. I like the new text from line 184 to 208 and is appropriate.

A good progress can be seen in revised manuscript. I think remaining repeating information and consistency of the text in the manuscript can take care by the editor with the help of the authors at the editorial process.

Response : Thanks for the feedback on the revised manuscript.

**# Additional comments**

Throughout this manuscript uses the words ‘envenomation’ and ‘envenoming’ interchangeably. It looks authors have not given much attention to the difference between these 2 words. I think if the bite is an accident, then the best word would be the envenoming that WHO uses https://www.who.int/snakebites/disease/en/. Also I see another word “anti-snake venom”. The correct word should be the “anti-venom”. Refer same WHO and recent leading research articles on Snakebites.

Response: Thank you for the suggestion and sharing the link of WHO reference document for snakebite. In the revised manuscript, we have used the word envenoming and deleted the word envenomation.

Similarly, the word “anti-snake venom” is replaced with “anti-venom” in the revised manuscript.
