# Supplementary material for: Perceptions, awareness on snakebite envenoming among the tribal community and health care providers of Dahanu block, Palghar District in Maharashtra, India
Source: PLoS One. 2021 Aug 5;16(8):e0255657. doi: 10.1371/journal.pone.0255657 (PMC8341635; doi:10.1371/journal.pone.0255657)
Supplement: Attachment — Submitted filename: Resposne to reviewers.docx [file pone.0255657.s013.docx]

**Response to Reviewer’s comments**

Journal Requirements:

Please review your reference list to ensure that it is complete and correct. If you have cited papers that have been retracted, please include the rationale for doing so in the manuscript text, or remove these references and replace them with relevant current references. Any changes to the reference list should be mentioned in the rebuttal letter that accompanies your revised manuscript. If you need to cite a retracted article, indicate the article’s retracted status in the References list and also include a citation and full reference for the retraction notice.

Response:

We reviewed the references and confirm that the reference list is complete and correct. During the revision of the manuscript, the following references were deleted

Reference no. 1,2,7,8, 33,38

In the revised manuscript, the following references are added

7. Sharma SK, Chappuis F, Jha N, Bovier PA, Loutan L, Koirala S. Impact of snake bites and determinants of fatal outcomes in southeastern Nepal. Am J Trop Med Hyg. 2004;71: 234–238.

31. Dey A, De JN. Traditional use of plants against snakebite in Indian subcontinent: a review of the recent literature. Afr J Tradit Complement Altern Med. 2011;9: 153–174. doi:10.4314/ajtcam.v9i1.20

Reviewer #3: I am glad to see a reasonable progress in revised manuscript. Thanks, authors have well responded to my initial comments and no any technical issues from me.

Even though I said no issues from me, manuscript needs a significant language improvement, condense the long descriptions squeeze into shorter, and eliminate all remaining repetitions and unrelated texts before publishing.

It is a serious challenge for editor and authors but would not be a big issue if you work seriously. Good luck

Response: As per the comments, we have edited the manuscript for language improvement. Also condensed the long sentences into shorter ones and removed the repetitions and unrelated text in the revised manuscript. We hope that the revised version of our manuscript is acceptable for publication.
